# Supplementary material for: Hyperinsulinemic and Pro-Inflammatory Dietary Patterns and Metabolomic Profiles Are Associated with Increased Risk of Total and Site-Specific Cancers among Postmenopausal Women
Source: Cancers (Basel). 2023 Mar 14;15(6):1756. doi: 10.3390/cancers15061756 (PMC10046106; doi:10.3390/cancers15061756)
Supplement: Supplementary file 1 [file cancers-15-01756-s001.zip › cancers-2251828-supplementary.pdf]

Table of contents

Supplementary Figure S1. Workflow to derive the final analytic datasets. Total cancer, colorectal cancers, invasive breast cancer, and lung cancer had 0 participants with no cancer outcome exposure. HEI-2015 analyses in total cancer excluded extra 2 participants without HEI-2015 diet information.
Supplementary Table S1. Food group components of the empirical dietary index for hyperinsulinemia (EDIH) score, empirical dietary inflammatory pattern (EDIP) score and Health Eating Index (HEI) 2015.
Supplementary Table S2. Cancer sites definitions.
Supplementary Table S3. Description of covariates used in the current study.
Supplementary Table S4. Distribution of all nutrients available in the WHI by score quintiles in the total cancer analytic dataset.
Supplementary Table S5. Hazard ratios (95% CI) for the associations of dietary patterns with total and site-specific cancers, further adjusted for body mass index and type 2 diabetes.
Supplementary Table S6. Multivariable-adjusted associations of dietary patterns with specific cancers in body mass index (kg/m2) subgroups.
Supplementary Table S7. Multivariable-adjusted associations of dietary patterns with specific cancers in type 2 diabetes subgroups.
Supplementary Table S8. Multivariable-adjusted associations of dietary patterns with specific cancers including mutual adjustment

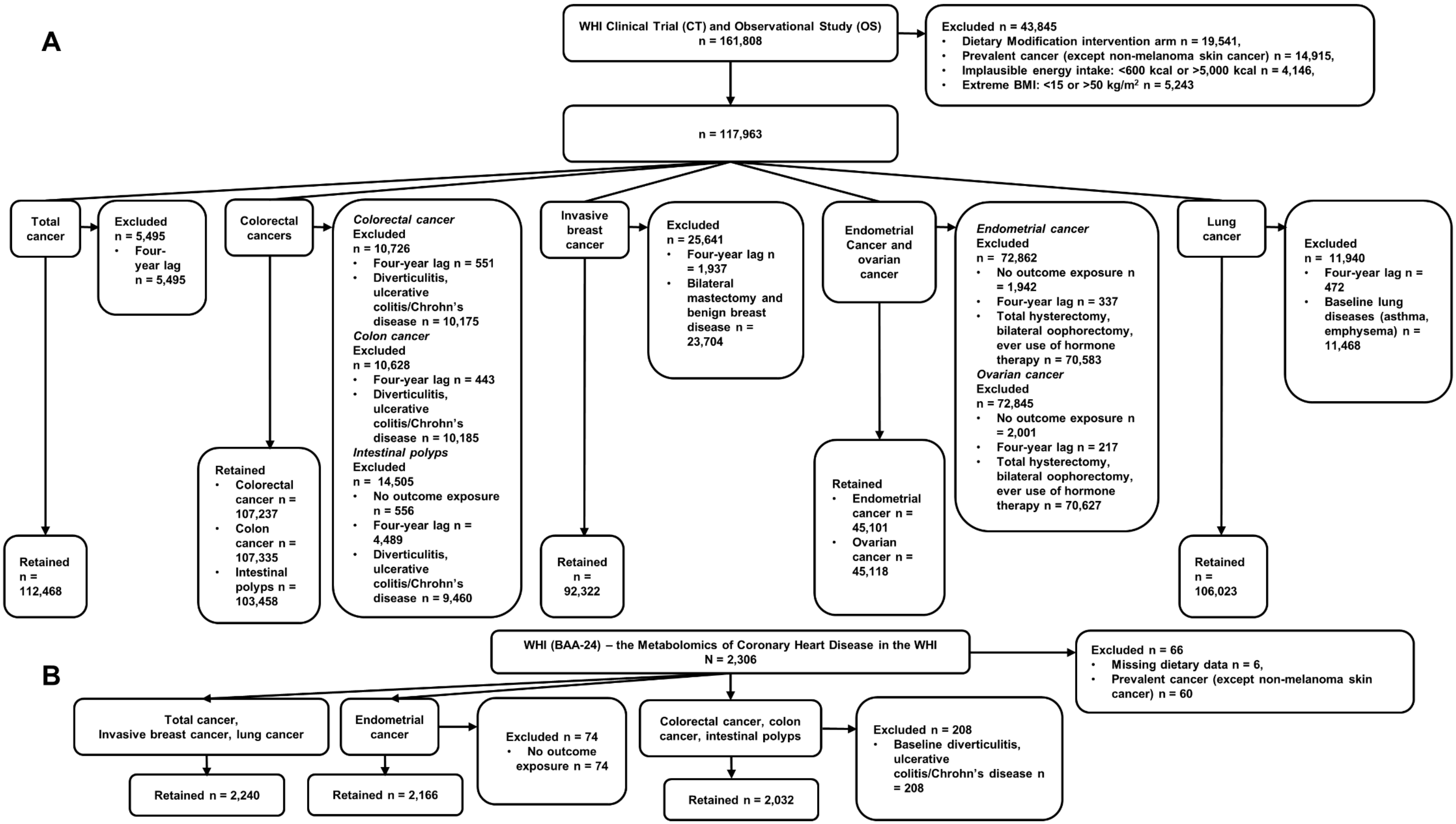

Supplementary Figure S1. Workflow to derive the final analytic datasets. A.The WHI dataset used to analyze dietary patterns in relation to total cancer and site-specific cancer risks. Total cancer, colorectal cancers, invasive breast cancer, and lung cancer had 0 participants with no cancer outcome exposure. HEI-2015 analyses in total cancer excluded extra 2 participants without HEI-2015 diet information. B. The metabolomics dataset used to analyze dietary pattern-related metabolomics signatures in relation to total cancer and site-specific cancer risks.

Supplementary Table S1. Food group components of the empirical dietary index for hyperinsulinemia (EDIH) score, empirical dietary inflammatory pattern (EDIP) score and Health Eating Index (HEI) 2015.

EDIH components	Weight	Food items
		Positive components
Processed meat	0.199	Processed meats (lunch meat other lunch meat) , bacon, hot dog
Red meat	0.25	Beef, pork and lamb as a main dish , ground meat incl hamburgers, Beef, pork, and lamb as a sandwich, stew, pot pie and casseroles with meat, gravies made with meat drippings, Menudo and tortilla soup
Low-energy sugary beverages	0.053	Low-energy cola, other low-energy carbonated beverages
High-energy sugary beverages	0.104	Regular soft drinks (not diet)
Margarine	0.054	Margarine
Butter	0.094	Butter
French fries	0.581	French fries
Non-dark fish	0.172	Tuna, shrimp, lobster, scallops, seafood other than dark fish
Eggs	0.124	Egg
Low-fat dairy	0.025	Low-fat milk , sherbet or ice milk, yogurt, low-fat desserts
Cream soup	0.787	Chowder or cream soup
Tomatoes	0.095	Fresh tomato & tomato juice, tomato sauce
Poultry	0.183	Chicken & turkey , fried chicken, Chicken or turkey with or without skin
		Inverse components
Green leafy vegetables	-0.055	Spinach& mustard greens& turnip greens& collards, iceberg or head lettuce, romaine or leaf lettuce
Wine	-0.165	Red, white wine
Coffee	-0.035	Coffee (regular or decaffeinated)
High-fat diary	-0.046	Whole milk , cream , sour cream ice cream, cream cheese, other cheese
Whole fruit	-0.029	Raisins, grapes, avocado, banana, cantaloupe, watermelon, orange, apple, pear, grapefruit, strawberries, blueberries, peaches, apricots, plums
**EDIP components**	**Weight**	**Food items**
		Positive components
Processed meat	165.03	Hot dogs, processed meats (including processed meat sandwich) , bacon
Red meat	140.19	Hamburger, beef /pork /lamb sandwich, beef /pork/ lamb main dish
Organ meat	144.61	Livers
Other fish	252.45	Canned tuna, shrimp, breaded fish, lobster, scallops or other seafood
Other vegetables	136.14	Corn, mixed vegetables, eggplant, celery, alfalfa sprouts, mushrooms, green/yellow/red peppers, zucchini, cucumbers
Refined grain	81.21	White bread, white rice, bagels/English muffins/rolls, muffins or biscuits, pasta, pancakes or waffles, refined cold breakfast cereals
High energy beverage	156.85	Cola, Hawaiian punch, caffeine-free coke, pepsi, carbonated beverage with caffeine and sugar, other carbonated beverage with sugar

Low energy beverage	94.77	Low calorie cola, low calorie caffeine -free cola, low calorie beverage with caffeine, other low calorie carbonated beverage, other low calorie beverage without caffeine
Tomato	167.92	Fresh tomatoes, tomato juice, tomato sauce
		Negative components
Beer	−136.99	Beer, light beer
Wine	−249.70	White wine, red wine
Tea	−42.25	Tea, tea (not herbal)
Coffee	−83.18	Coffee, decaffeinated coffee
Dark yellow vegetable	−165.37	Carrots, sweet potatoes, winter squash
Green leafy vegetable	−190.29	Spinach, iceberg lettuce, romaine lettuce
Snack	−45.08	Potato/corn chips, popcorn, crackers
Fruit juice	−58.95	Apple juice, orange juice, grape juice, prune juice, other juice
Pizza	−1175.21	Pizza

**HEI2015 components**	**Maximum points**
Adequacy	
Total Fruits	5
Whole Fruits	5
Total Vegetables	5
Greens and Beans	5
Whole Grains	10
Dairy	10
Total Protein Foods	5
Seafood and Plant Proteins	5
Fatty Acids	10
Moderation	
Refined Grains	10
Sodium	10
Added Sugars	10
Saturated Fats	10

Supplementary Table S2. Cancer sites definitions

Cancer sites	Descriptions
Total cancer	The first occurrence of any cancer except non-melanoma skin cancer
Colorectal cancer subtypes	Proximal colon cancer was defined based on the anatomic subsites, cecum, ascending colon, hepatic flexure of colon, and transverse colon (ICD site codes 18.0,18.2,18.3, 18.4)
	Distal colon cancer was defined as cancers of splenic flexure of colon, descending colon, sigmoid colon (ICD site codes 18.5, 18.6,18.7)
Breast cancer subtypes	Breast cancer subtypes were defined based on the status of estrogen receptor (ER), progesterone receptor (PR), human epidermal growth factor receptor (HER2),[a] and were combined to define triple negative (ER- PR- HER2-), luminal A (ER+ and/or PR+, HER2-), luminal B (ER+ and/or PR+, HER2+)
	Other subtypes were defined based on Surveillance Epidemiology and End Results program (SEER) histology codes, including invasive ductal carcinoma (8500/3) and invasive lobular carcinoma (8520/3).
Endometrial cancer subtypes	Endometrioid (8140/3,8380/3); non-endometrioid - cases other than endometrioid cases.
Ovarian cancer subtypes	Serous ovarian cancer (8020/3, 8021/3, 8022/3, 8050/3, 8120/3, 8130/2, 8130/3, 8260/3, 8441/1, 8441/3, 8442/1, 8442/3, 8450/3, 8460/3, 8461/3, 8462/1, 9014/3)
	Non-serous ovarian cancer (8380/3, 8381/3, 8382/3, 8383/3, 8310/3, 8470/3, 8471/3, 8472/1, 8480/3, 8950/3, 8323/3, 9000/1, 9000/3)
Lung cancer subtypes	Small-cell lung cancer - (8041/3, 8042/3,8043/3,8044/3, 8045/3)[b]
	Non-small cell lung cancer (8046/3, 8070/3, 8140/3, 8250/3, 8012/3, 8560/3, 8240/3, 9050/3)

a.Curb JD, McTiernan A, Heckbert SR, Kooperberg C, Stanford J, Nevitt M, Johnson KC, Proulx-Burns L, Pastore L, Criqui M, Daugherty S; WHI Morbidity and Mortality Committee. Outcomes ascertainment and adjudication methods in the Women's Health Initiative. Ann Epidemiol. 2003 Oct;13(9 Suppl):S122-8. doi: 10.1016/s1047-2797(03)00048-6. PMID: 14575944.

b.SEER Training Modules, Lung Cancer, Abstracting, Coding, & Staging, Morphology & Grade. U. S. National Institutes of Health, National Cancer Institute

Supplementary Table S3. Description of covariates used in the current study

Covariates	Variable Description
Total energy intake	Dietary energy intake (kcal/day)
	**Covariates included in all models**
Age, years	Age at screening, <50-59, 60-69, 70-79+
Physical activity	Total energy expended from recreational physical activity (MET-hours/week)
Self-reported race and ethnicity	1 American Indian or Alaskan Native; 2 Asian or Pacific Islander; 3 Black or African American; 4 Hispanic/Latino; 5 White (not of Hispanic origin);8 Other
Education	Didn't go to school, grade school (1-8 yrs); Some high school (9-11 yrs)/High school diploma/GED/Vocational or training school/Some college or associate degree; Some post-graduate or professional/College graduate or Baccalaureate Degree/Master's Degree/Doctoral Degree (Ph.D,M.D.,J.D.,etc.). Missing values were imputed by income
Family history of cancer	Yes/no
Hormone use	Number of hormones used. The variable was created from a sum of the following 8 WHI variables: Oral contraceptive use ever, diethylstilbestrol use ever, depo-provera use ever, unopposed estrogen use ever, Estrogen + progesterone use ever, Testosterone or other male hormone use, Estratest use, oral daily use of a glucocorticosteroid
Supplement	Number of supplements taken. The variable was created from a sum of the following 23 supplements variables (yes=1/no=0): vitamin A, alpha-tocopherol, vitamin B1, vitamin B12, vitamin B2, vitamin B6, beta-carotene, biotin, vitamin C, calcium, chromium, copper, vitamin D, folic acid, iron, magnesium, manganese, molybdenum, niacin, pantothenic acid, retinol, selenium, and zinc
Comorbidity score	liver disease, lost 15lb past 6mo, dialysis, other chronic disease, stomach ulcer, diverticulitis, colitis, pancreatitis, arthritis, rheumatoid arthritis, gallstone or gallbladder disease, hypertension, high cholesterol
Baseline cardiovascular disease status	Yes/no
Baseline lung disease	Asthma, emphysema, or both
Pack-years of smoking	Continuous variable
Hormone Replacement Therapy (HT) study arm	HT study arm to which the participant was randomized: Not randomized to HRT;Estrogen-alone intervention; Estrogen-alone control;Estrogen + Progestin intervention;Estrogen+Progestin control
NSAID	Baseline nonsteroidal anti-inflammatory agents use: no=1, yes=1
Baseline hormone therapy ever	Yes/no
Oral contraceptive duration	years
Coffee/tea	Coffee or tea (all types), med serv/day
Total alcohol intake	Alcohol servings per week (wine, beer and liquor servings). Number of servings per week of beer, wine and/or liquor based on a medium serving size which is 12oz of beer, 6oz of wine and 1.5 oz of liquor.
	**Covariates only included in colorectal cancer and subtype analyses**
Colorectal cancer screening	Hemoccult test or colonoscopy ever, yes/no
**Covariates further included in breast cancer and subtype analyses**	
Months of breast-feeding	Never breastfed, 1-6 months,7-12 Months, 13-23 Months, 24+ Months
Age at menopause	Continuous variable, age at which participant went through menopause
Mammogram ever	Yes/no

Parity	Number of Term Pregnancies: never pregnant, never had term pregnancy, 1,2,3,4,5+
Bilateral oophorectomy	Yes/no
Passive smoking	Yes/no
Gail 5-year risk score	Continuous
**Covariates further included in endometrial and ovarian cancer and subtype analyses**	
Age at first birth	Never had term pregnancy,< 20,20-29, 30+
Age at menarche	9 or less, 10,11,12,13,14,15,16,17 or older
Age at menopause	Continuous variable, age at which participant went through menopause
Months of breast-feeding	Never breastfed, 1-6 months,7-12 Months, 13-23 Months, 24+ Months
Parity	Number of Term Pregnancies: never pregnant, never had term pregnancy, 1,2,3,4,5+
Tubal ligation (only for ovarain cancer analyses)	Yes/no
**Covariates further included in lung cancer and subtype analyses**	
Passive smoking	Yes/no
Smoking status	Never Smoked,past smoker,current smoker
**Other covariates**	
Body mass index (BMI)	Continuous, kg/m^2
Diabetes ever	Yes/no

Supplementary Table S4. Distribution of all nutrients available in the WHI by score quintiles in the total cancer analytic dataset [a]

	Empirical Index for Hyperinsulinemic (EDIH) score					Empirical Inflammatory Pattern (EDIP) score					Health Eating Index 2015 (HEI-2015)				
	Quintile 1	Quintile 2	Quintile 3	Quintile 4	Quintile 5	Quintile 1	Quintile 2	Quintile 3	Quintile 4	Quintile 5	Quintile 1	Quintile 2	Quintile 3	Quintile 4	Quintile 5
	(-10.52, -0.80)	(-0.80, -0.27)	(-0.27, 0.16)	(0.16, 0.67)	(0.67, 8.89)	(-11.59, -0.75)	(-0.75, -0.16)	(-0.16, 0.28)	(0.28, 0.73)	(0.73, 6.55)	(-4.33, -0.81)	(-0.81, -0.16)	(-0.16, 0.37)	(0.37, 0.92)	(0.92,3.06)
**Carbohydrates** [b]															
Total Carbohydrate	135.91±24.52	134.88±22.32	130.16±21.18	122.42±19.62	112.09±21.26	126.19±24.66	128.30±23.34	128.47±22.96	127.82±22.98	124.56±23.67	114.31±22.21	120.23±21.91	126.8±21.92	133.15±21.6	140.95±20.49
Pectins	1.87±0.89	1.86±0.89	1.75±0.85	1.53±0.73	1.18±0.55	1.81±0.88	1.78±0.83	1.71±0.83	1.61±0.81	1.29±0.7	0.97±0.46	1.36±0.60	1.65±0.73	1.95±0.82	2.26±0.83
Starch	49.27±13.46	49.48±12.58	48.92±12.09	47.76±11.56	44.69±10.86	46.79±12.4	47.86±11.79	48.27±11.72	48.74±12.14	48.48±13.17	47.89±13.1	47.72±12.69	47.95±12.48	48.06±11.98	48.52±10.99
Total fiber	11.55±3.91	11.45±3.73	10.9±3.54	9.86±3.06	8.08±2.53	10.89±3.76	10.89±3.57	10.68±3.57	10.33±3.53	9.05±3.37	7.28±2.16	9.02±2.56	10.36±2.96	11.75±3.30	13.43±3.50
Water soluble fiber	3.05±1.02	3.04±0.99	2.90±0.93	2.64±0.80	2.22±0.65	2.87±0.98	2.89±0.94	2.85±0.94	2.77±0.92	2.46±0.85	2.10±0.61	2.46±0.72	2.76±0.83	3.08±0.91	3.46±0.96
Insoluble fiber	8.45±3.02	8.37±2.88	7.96±2.75	7.16±2.38	5.80±1.98	7.97±2.90	7.95±2.76	7.78±2.76	7.51±2.74	6.53±2.63	5.12±1.66	6.50±1.97	7.55±2.27	8.63±2.54	9.95±2.69
Total Sugars	67.21±18.81	66.52±17.99	63.43±17.5	58.56±16.93	54.01±20.97	60.84±18.95	62.53±18.22	62.79±18.24	62.37±18.52	61.20±21.57	53.73±20.29	57.28±18.5	61.65±18.03	65.92±17.4	71.15±16.22
Galactose	0.25±0.27	0.23±0.24	0.20±0.21	0.18±0.20	0.14±0.15	0.23±0.24	0.22±0.23	0.21±0.22	0.19±0.22	0.16±0.19	0.15±0.18	0.19±0.22	0.21±0.23	0.22±0.23	0.24±0.23
Glucose	14.34±5.66	14.4±5.68	13.91±5.66	12.95±5.54	12.82±7.62	13.28±5.5	13.72±5.6	13.74±5.68	13.68±5.89	14.01±7.65	11.35±6.81	12.51±5.94	13.78±5.75	14.86±5.56	15.92±5.34
Fructose	14.1±6.4	14.26±6.48	13.85±6.56	12.94±6.53	13.32±9.66	13.05±6.25	13.54±6.37	13.61±6.52	13.64±6.85	14.62±9.63	11.09±8.5	12.31±7.10	13.71±6.72	15.00±6.42	16.36±6.05
Lactose	12.7±8.42	12.27±8.08	11.31±7.64	10.07±6.96	8.04±5.72	11.02±7.77	11.25±7.38	11.30±7.56	11.08±7.80	9.73±7.44	7.90±6.08	9.45±6.76	10.60±7.36	11.96±7.72	14.47±8.27
Maltose	2.08±0.93	1.97±0.85	1.85±0.80	1.72±0.73	1.52±0.64	1.94±0.91	1.9±0.83	1.86±0.8	1.80±0.79	1.66±0.73	1.59±0.69	1.70±0.76	1.82±0.80	1.93±0.85	2.11±0.90
Sucrose	23.74±9.14	23.39±8.41	22.32±7.85	20.71±7.51	18.17±6.87	21.33±8.83	21.91±8.17	22.07±8.05	21.98±8.08	21.02±8.05	21.64±9.85	21.12±8.45	21.54±8.04	21.96±7.63	22.07±6.97
Added Sugars	28.96±12.06	28.78±11.52	28.33±11.69	28.23±12.8	31.31±19.95	26.27±11.42	27.47±11.29	28.21±11.66	29.32±12.69	34.35±19.72	34.89±18.87	30.15±14.43	28.57±12.66	27.01±10.85	25.01±9.21
**Protein** [b]															
Total Protein	39.84±7.37	41.4±7.49	42.15±7.52	43.16±8.03	43.45±9.12	41.71±7.99	42.27±7.68	42.28±7.7	42.24±7.89	41.49±8.84	39.00±8.25	41.29±7.76	42.26±7.7	43.23±7.84	44.21±7.62
Animal Protein	25.77±8.11	27.69±8.07	29.02±7.91	30.86±8.21	32.66±9.09	28.45±8.58	29.10±8.38	29.29±8.37	29.53±8.54	29.62±9.19	28.24±8.72	29.44±8.43	29.54±8.52	29.59±8.72	29.19±8.68

Vegetable Protein	14.02±4.01	13.65±3.5	13.08±3.18	12.24±2.85	10.73±2.57	13.18±3.56	13.11±3.35	12.93±3.34	12.67±3.35	11.83±3.53	10.70±2.82	11.79±2.92	12.66±3.09	13.59±3.23	14.97±3.57
Alanine	1.79±0.37	1.9±0.38	1.98±0.39	2.07±0.43	2.15±0.51	1.94±0.43	1.98±0.42	1.98±0.42	2.00±0.43	2±0.49	1.86±0.47	1.97±0.44	2.00±0.43	2.03±0.43	2.04±0.40
Arginine	2±0.39	2.12±0.4	2.20±0.41	2.3±0.46	2.39±0.55	2.15±0.45	2.20±0.44	2.21±0.44	2.22±0.46	2.23±0.53	2.08±0.51	2.19±0.47	2.22±0.45	2.26±0.45	2.27±0.41
Aspartic Acid	3.45±0.67	3.61±0.68	3.69±0.68	3.79±0.74	3.84±0.84	3.64±0.73	3.7±0.71	3.7±0.70	3.70±0.72	3.63±0.82	3.34±0.76	3.59±0.71	3.71±0.70	3.82±0.71	3.92±0.68
Cystine	0.63±0.18	0.66±0.18	0.66±0.17	0.66±0.16	0.64±0.16	0.63±0.17	0.65±0.16	0.66±0.17	0.66±0.17	0.64±0.17	0.57±0.14	0.62±0.15	0.65±0.16	0.68±0.17	0.73±0.19
Glutamic Acid	8.04±1.43	8.23±1.4	8.27±1.39	8.33±1.43	8.20±1.56	8.2±1.47	8.30±1.38	8.28±1.39	8.25±1.41	8.05±1.57	7.66±1.46	8.07±1.39	8.25±1.39	8.44±1.41	8.65±1.39
Glycine	1.5±0.3	1.61±0.32	1.69±0.33	1.80±0.38	1.91±0.47	1.65±0.38	1.69±0.37	1.70±0.37	1.72±0.39	1.75±0.45	1.65±0.44	1.71±0.40	1.72±0.39	1.73±0.38	1.71±0.34
Histidine	1.05±0.21	1.11±0.21	1.14±0.22	1.19±0.24	1.23±0.28	1.12±0.24	1.15±0.23	1.15±0.23	1.15±0.24	1.15±0.27	1.09±0.26	1.14±0.24	1.15±0.24	1.17±0.24	1.17±0.22
Total BCAA	5.72±1.17	5.94±1.18	6.05±1.18	6.20±1.24	6.24±1.37	5.99±1.26	6.08±1.20	6.07±1.20	6.06±1.22	5.95±1.34	5.61±1.27	5.94±1.21	6.08±1.21	6.21±1.23	6.31±1.19
Leucine	3.18±0.68	3.3±0.68	3.36±0.67	3.43±0.69	3.45±0.75	3.31±0.7	3.37±0.68	3.37±0.68	3.37±0.69	3.31±0.75	3.10±0.70	3.29±0.67	3.36±0.68	3.44±0.70	3.53±0.69
Valine	2.11±0.43	2.18±0.43	2.20±0.42	2.25±0.44	2.25±0.48	2.19±0.45	2.22±0.43	2.21±0.43	2.20±0.43	2.15±0.48	2.03±0.45	2.16±0.43	2.21±0.43	2.26±0.44	2.31±0.42
Isoleucine	1.81±0.37	1.88±0.38	1.92±0.38	1.98±0.40	2.00±0.45	1.90±0.40	1.93±0.39	1.93±0.39	1.93±0.39	1.9±0.43	1.79±0.41	1.89±0.39	1.93±0.39	1.97±0.40	2.00±0.38
Lysine	2.64±0.63	2.79±0.65	2.88±0.64	3.00±0.69	3.10±0.77	2.84±0.69	2.89±0.67	2.90±0.67	2.90±0.69	2.87±0.76	2.70±0.72	2.86±0.68	2.91±0.68	2.96±0.69	2.99±0.67
Methionine	0.88±0.19	0.92±0.2	0.95±0.20	1.00±0.21	1.02±0.24	0.94±0.21	0.96±0.21	0.96±0.21	0.96±0.21	0.96±0.23	0.91±0.22	0.95±0.21	0.97±0.21	0.98±0.21	0.97±0.20
Phenylalanine	1.79±0.33	1.84±0.33	1.86±0.33	1.90±0.35	1.89±0.38	1.85±0.35	1.87±0.33	1.87±0.33	1.86±0.34	1.83±0.38	1.72±0.35	1.82±0.34	1.87±0.33	1.91±0.34	1.96±0.33
Proline	2.92±0.65	2.95±0.63	2.92±0.60	2.90±0.59	2.80±0.57	2.91±0.63	2.94±0.59	2.93±0.59	2.91±0.60	2.8±0.62	2.67±0.57	2.83±0.57	2.90±0.59	2.99±0.61	3.10±0.61
Serine	1.84±0.36	1.89±0.35	1.91±0.35	1.94±0.36	1.94±0.40	1.89±0.37	1.92±0.35	1.92±0.35	1.92±0.36	1.88±0.4	1.76±0.37	1.87±0.35	1.91±0.35	1.96±0.36	2.02±0.35
Threonine	1.44±0.29	1.51±0.3	1.55±0.3	1.61±0.33	1.65±0.38	1.53±0.33	1.56±0.32	1.56±0.31	1.56±0.32	1.55±0.36	1.46±0.35	1.54±0.33	1.57±0.32	1.59±0.32	1.60±0.31
Tryptophan	0.45±0.09	0.46±0.09	0.46±0.09	0.47±0.09	0.46±0.10	0.46±0.10	0.46±0.09	0.46±0.09	0.46±0.09	0.45±0.10	0.42±0.09	0.45±0.09	0.46±0.09	0.48±0.09	0.49±0.09
Tyrosine	1.43±0.31	1.47±0.31	1.49±0.30	1.52±0.31	1.52±0.33	1.48±0.32	1.50±0.3	1.50±0.30	1.49±0.31	1.46±0.33	1.38±0.31	1.46±0.30	1.50±0.30	1.53±0.31	1.56±0.31
3-Methylhistidine [c]	5.95±2.96	7.12±3.07	8.15±3.17	9.43±3.49	11.08±4.3	7.71±3.66	8.06±3.67	8.25±3.71	8.53±3.84	9.16±4.27	8.60±4.15	8.74±3.95	8.54±3.83	8.27±3.75	7.57±3.52
**Fats [b]**															
Total Fat	30.71±9.06	32.57±8.79	34.70±8.5	37.69±8.08	42.03±8.15	33.35±9.55	34.48±9.25	35.31±9.12	36.24±9.16	38.31±9.23	42.44±8.14	38.76±8.28	35.37±8.31	32.31±8.17	28.83±7.68
Cholesterol [c]	104.37±43.9	116.05±45.92	127.81±48.48	144.45±55.31	164.39±66.49	122.5±55.86	126.74±54.07	129.97±54.26	134.42±55.86	143.44±61.15	154.03±65.69	143.13±57.61	132.69±52.28	121.46±48.30	105.76±44.68

Total SFA	10.32±3.71	10.81±3.51	11.47±3.38	12.47±3.26	14.05±3.39	11.14±3.8	11.48±3.66	11.74±3.60	12.04±3.63	12.72±3.62	15.15±3.51	13.25±3.16	11.73±2.91	10.34±2.64	8.65±2.33
SFA 4:0	0.26±0.17	0.26±0.16	0.26±0.16	0.27±0.17	0.30±0.19	0.27±0.18	0.27±0.17	0.27±0.17	0.27±0.17	0.26±0.17	0.38±0.21	0.32±0.17	0.27±0.14	0.22±0.12	0.16±0.09
SFA 6:0	0.12±0.09	0.12±0.09	0.13±0.09	0.13±0.09	0.15±0.11	0.13±0.10	0.13±0.09	0.13±0.09	0.13±0.09	0.13±0.09	0.19±0.12	0.16±0.10	0.13±0.08	0.10±0.06	0.08±0.05
SFA 8:0	0.1±0.06	0.09±0.06	0.10±0.06	0.10±0.06	0.11±0.07	0.10±0.06	0.10±0.06	0.10±0.06	0.10±0.06	0.10±0.07	0.14±0.08	0.12±0.06	0.10±0.05	0.08±0.04	0.06±0.03
SFA 10:0	0.18±0.12	0.18±0.12	0.19±0.12	0.20±0.12	0.22±0.14	0.19±0.13	0.20±0.12	0.20±0.12	0.20±0.12	0.20±0.12	0.28±0.15	0.23±0.12	0.19±0.10	0.16±0.08	0.12±0.06
SFA 12:0	0.25±0.18	0.25±0.17	0.26±0.17	0.27±0.17	0.30±0.18	0.25±0.16	0.26±0.17	0.26±0.17	0.27±0.18	0.29±0.19	0.39±0.22	0.31±0.16	0.26±0.14	0.21±0.12	0.16±0.11
SFA 14:0	0.91±0.49	0.92±0.46	0.95±0.45	1.01±0.45	1.12±0.48	0.96±0.49	0.98±0.47	0.98±0.46	0.99±0.47	1.01±0.46	1.34±0.54	1.14±0.45	0.97±0.38	0.83±0.32	0.64±0.26
SFA 16:0, Palmitic Acid	5.47±1.77	5.79±1.68	6.18±1.62	6.75±1.55	7.63±1.62	5.98±1.85	6.17±1.78	6.31±1.76	6.49±1.78	6.88±1.80	7.94±1.67	7.07±1.55	6.33±1.46	5.66±1.36	4.82±1.23
SFA 17:0	0.03±0.02	0.03±0.02	0.04±0.02	0.04±0.03	0.05±0.03	0.04±0.03	0.04±0.02	0.04±0.03	0.04±0.03	0.04±0.03	0.05±0.03	0.05±0.03	0.04±0.02	0.03±0.02	0.02±0.02
SFA 18:0, Stearic Acid	2.63±0.99	2.81±0.95	3.03±0.91	3.34±0.86	3.84±0.86	2.87±1.00	2.99±0.97	3.09±0.97	3.21±0.99	3.47±1.00	4.09±0.86	3.54±0.81	3.10±0.78	2.71±0.73	2.21±0.67
SFA 20:0	0.05±0.04	0.05±0.03	0.05±0.03	0.05±0.03	0.05±0.03	0.05±0.03	0.05±0.03	0.05±0.03	0.05±0.03	0.05±0.03	0.05±0.03	0.05±0.03	0.05±0.03	0.05±0.03	0.05±0.04
SFA 22:0	0.06±0.07	0.05±0.06	0.05±0.05	0.05±0.05	0.04±0.04	0.05±0.06	0.05±0.06	0.05±0.05	0.05±0.05	0.04±0.05	0.04±0.04	0.04±0.05	0.05±0.05	0.05±0.06	0.06±0.07
Total MFA	11.52±3.74	12.24±3.68	13.11±3.57	14.32±3.40	16.11±3.37	12.60±3.95	13.04±3.86	13.36±3.79	13.72±3.78	14.60±3.85	15.93±3.39	14.64±3.52	13.41±3.60	12.28±3.58	11.05±3.46
MFA 14:1	0.02±0.02	0.03±0.02	0.03±0.02	0.03±0.02	0.04±0.02	0.03±0.02	0.03±0.02	0.03±0.02	0.03±0.02	0.04±0.02	0.04±0.02	0.03±0.02	0.03±0.02	0.03±0.02	0.02±0.01
MFA 16:1	0.44±0.16	0.48±0.16	0.53±0.16	0.60±0.16	0.70±0.18	0.51±0.18	0.53±0.18	0.54±0.18	0.56±0.18	0.61±0.20	0.68±0.20	0.61±0.17	0.55±0.16	0.49±0.14	0.41±0.13
MFA 18:1, Oleic Acid	10.63±3.51	11.31±3.46	12.11±3.38	13.25±3.24	14.92±3.21	11.64±3.72	12.05±3.65	12.35±3.58	12.68±3.58	13.5±3.64	14.66±3.21	13.52±3.34	12.40±3.43	11.37±3.43	10.27±3.32
MFA 20:1	0.09±0.06	0.10±0.05	0.10±0.05	0.11±0.05	0.12±0.04	0.10±0.06	0.10±0.05	0.10±0.05	0.10±0.05	0.11±0.05	0.11±0.05	0.10±0.05	0.10±0.05	0.10±0.05	0.10±0.06
MFA 22:1	0.01±0.01	0.01±0.01	0.01±0.01	0.01±0.01	0.01±0.01	0.01±0.01	0.01±0.01	0.01±0.01	0.01±0.01	0.01±0.01	0.01±0.01	0.01±0.01	0.01±0.01	0.01±0.01	0.01±0.01
Total PFA	6.49±2.24	6.95±2.27	7.36±2.33	7.87±2.35	8.42±2.40	6.97±2.35	7.23±2.32	7.41±2.35	7.58±2.39	7.9±2.55	8.02±2.39	7.79±2.44	7.39±2.39	7.09±2.39	6.80±2.25
Omega 3 PFA	0.8±0.32	0.84±0.32	0.87±0.32	0.90±0.31	0.93±0.3	0.86±0.34	0.87±0.32	0.87±0.31	0.87±0.31	0.87±0.31	0.88±0.29	0.89±0.31	0.87±0.32	0.86±0.33	0.86±0.33
Omega 6 PFA	5.65±2.03	6.06±2.05	6.44±2.10	6.90±2.12	7.40±2.15	6.06±2.11	6.31±2.09	6.48±2.12	6.65±2.16	6.96±2.30	7.07±2.15	6.83±2.19	6.46±2.15	6.17±2.15	5.90±2.04
PFA 18:2, Linoleic Acid	5.65±2.03	6.06±2.05	6.44±2.1	6.89±2.12	7.40±2.15	6.05±2.11	6.30±2.09	6.48±2.12	6.65±2.16	6.95±2.30	7.07±2.15	6.83±2.19	6.46±2.15	6.17±2.15	5.90±2.04
PFA 18:3, Linolenic Acid	0.72±0.30	0.75±0.3	0.78±0.3	0.81±0.3	0.84±0.29	0.77±0.32	0.78±0.30	0.78±0.30	0.78±0.29	0.79±0.30	0.81±0.28	0.80±0.30	0.77±0.30	0.76±0.31	0.75±0.31
PFA 18:4	0.00±0.00	0.00±0.00	0.00±0.00	0.00±0.00	0.00±0.00	0.00±0.00	0.00±0.00	0.00±0.00	0.00±0.00	0.00±0.00	0.00±0.00	0.00±0.00	0.00±0.00	0.00±0.00	0.00±0.00

PFA 20:4	0.05±0.02	0.05±0.03	0.06±0.03	0.07±0.03	0.08±0.04	0.06±0.03	0.06±0.03	0.06±0.03	0.06±0.03	0.07±0.03	0.06±0.03	0.06±0.03	0.06±0.03	0.06±0.03	0.05±0.03
PFA 20:5, EPA	0.03±0.03	0.03±0.03	0.03±0.03	0.03±0.03	0.03±0.03	0.03±0.03	0.03±0.03	0.03±0.03	0.03±0.03	0.03±0.03	0.02±0.02	0.03±0.02	0.03±0.03	0.03±0.03	0.03±0.03
PFA 22:5	0.01±0.01	0.01±0.01	0.01±0.01	0.01±0.01	0.01±0.01	0.01±0.01	0.01±0.01	0.01±0.01	0.01±0.01	0.01±0.01	0.01±0.01	0.01±0.01	0.01±0.01	0.01±0.01	0.01±0.01
PFA 22:6, dha	0.05±0.06	0.06±0.06	0.05±0.05	0.05±0.05	0.05±0.05	0.06±0.06	0.06±0.05	0.05±0.05	0.05±0.05	0.05±0.05	0.04±0.04	0.05±0.05	0.05±0.05	0.06±0.06	0.07±0.06
Total Trans Fatty Acid	1.97±1.05	2.19±1.12	2.42±1.18	2.73±1.25	3.16±1.30	2.23±1.19	2.38±1.23	2.48±1.24	2.59±1.26	2.79±1.27	3.11±1.23	2.78±1.21	2.49±1.21	2.22±1.16	1.88±1.08
Trans Fatty Acid, 161T	0.02±0.02	0.02±0.02	0.02±0.02	0.03±0.02	0.04±0.03	0.02±0.02	0.02±0.02	0.02±0.02	0.03±0.02	0.03±0.02	0.04±0.03	0.03±0.02	0.02±0.02	0.02±0.01	0.01±0.01
Trans Fatty Acid, 181T	1.69±0.95	1.9±1.02	2.11±1.08	2.39±1.16	2.78±1.21	1.93±1.09	2.07±1.13	2.17±1.14	2.27±1.16	2.45±1.17	2.71±1.14	2.43±1.12	2.18±1.11	1.94±1.07	1.64±0.99
Trans Fatty Acid, 182T	0.23±0.11	0.25±0.11	0.26±0.11	0.29±0.10	0.32±0.10	0.25±0.11	0.26±0.11	0.27±0.11	0.28±0.11	0.30±0.11	0.34±0.10	0.30±0.10	0.27±0.10	0.24±0.10	0.20±0.09
Water	1205.2±400.57	1115±359.16	1029.63±316.97	937.65±285.66	820.45±262.19	1329.09±428.72	1114.40±295.12	1006.34±254.87	893.33±234.19	764.78±231.86	888.59±361.09	958.67±343.31	1026.78±343.01	1089.44±342.57	1144.46±325.01
Alcohol	6.43±9.68	3.63±5.98	2.87±5.50	2.36±4.97	1.77±4.08	7.05±9.96	4.06±6.13	2.89±5.23	1.96±4.33	1.09±3.20	2.31±5.28	3.02±6.05	3.62±6.89	3.95±7.06	4.15±7.09
Alcohol servings per week	2.85±4.31	1.65±2.8	1.33±2.65	1.11±2.44	0.85±2.03	3.17±4.49	1.86±2.88	1.34±2.51	0.92±2.11	0.52±1.58	1.07±2.50	1.39±2.83	1.66±3.18	1.80±3.24	1.88±3.25
Caffeine [c]	149.24±113.61	127.81±102.64	110.2±90.08	95.12±80.53	74.65±66.88	195.78±130.70	134.61±81.44	105.19±65.46	75.03±54.14	46.41±40.70	119.03±105.35	112.55±96.73	111.97±94.64	110.57±92.76	102.90±87.64
**Vitamins and Minerals**															
Vitamin A (mcg RE/d/1000kcal)	715.11±324.11	720.93±319.73	699.97±313.2	656.8±282.89	570.17±255.31	728.78±344.64	708.01±302.57	689.37±295.22	662.03±283.37	574.79±272.15	506.73±235.24	606.05±264.23	672.23±287.55	746.17±307.84	831.80±317.67
Retinol [d]	251.53±137.04	264.48±147.24	271.1±148.47	277.3±164.05	276.01±171.64	250.90±141.63	262.84±139.86	271.23±142.83	278.35±155.46	277.09±186.05	263.97±161.24	269.99±160.5	266.36±162.03	266.06±146.07	274.04±141.10
Thiamin [c]	0.88±0.22	0.9±0.22	0.89±0.23	0.85±0.20	0.78±0.18	0.86±0.21	0.87±0.21	0.87±0.22	0.87±0.22	0.82±0.22	0.78±0.18	0.83±0.20	0.86±0.22	0.90±0.22	0.94±0.22
Riboflavin [c]	1.35±0.39	1.33±0.38	1.28±0.37	1.21±0.33	1.07±0.29	1.37±0.39	1.30±0.35	1.26±0.35	1.22±0.36	1.10±0.35	1.12±0.32	1.19±0.34	1.25±0.37	1.30±0.38	1.38±0.39
Niacin [c]	10.79±2.95	11.33±3.19	11.53±3.36	11.46±3.14	10.97±2.91	11.27±3.02	11.36±3.05	11.34±3.15	11.27±3.22	10.86±3.19	10.14±2.67	10.87±2.91	11.35±3.17	11.71±3.21	12.02±3.30
Niacin Equivalents [c]	18.23±3.68	19.00±3.95	19.28±4.14	19.32±4.09	18.74±4.15	18.94±3.93	19.12±3.89	19.09±3.96	19.00±4.05	18.42±4.24	17.25±3.63	18.39±3.74	19.08±3.97	19.65±4.03	20.21±4.07
Pantothenic Acid [c]	3.65±1.39	3.62±1.45	3.49±1.51	3.24±1.28	2.83±1.04	3.80±1.36	3.55±1.31	3.42±1.35	3.23±1.38	2.82±1.27	2.83±1.09	3.15±1.23	3.40±1.39	3.61±1.42	3.84±1.48
Vitamin B6 [c]	1.03±0.33	1.08±0.36	1.08±0.37	1.03±0.33	0.93±0.28	1.03±0.32	1.05±0.33	1.06±0.34	1.04±0.35	0.96±0.34	0.80±0.24	0.94±0.28	1.04±0.31	1.13±0.33	1.22±0.35
Vitamin B12 [d]	3.32±1.48	3.57±1.61	3.72±1.65	3.85±1.76	3.98±1.94	3.40±1.51	3.57±1.52	3.69±1.58	3.83±1.72	3.95±2.10	3.41±1.78	3.66±1.75	3.74±1.73	3.78±1.65	3.85±1.60

Natural Folate (food folate/d/1000kcal) [d]	168.19±49.9	165.14±47.32	156.85±44.47	143.6±39.51	120.39±34.62	170.72±48.89	161.78±44.66	154.38±43.11	145.09±41.92	122.21±38.83	113.17±33.04	135.76±36.37	152.31±40.53	167.86±43.59	185.08±43.82
Vitamin C [c]	73.91±39.79	75.63±40.84	71.85±39.24	62.84±34.63	48.6±27.73	69.94±38.49	71.38±38.33	70.21±38.86	67.08±38.41	54.22±33.52	40.71±26.28	56.69±31.35	68.56±35.58	78.57±38.26	88.30±38.35
Vitamin D d	2.8±1.77	2.86±1.72	2.80±1.63	2.69±1.50	2.41±1.26	2.59±1.60	2.72±1.57	2.79±1.58	2.82±1.63	2.65±1.58	2.14±1.26	2.45±1.40	2.67±1.53	2.91±1.63	3.39±1.80
Vitamin E (IU/d/1000kcal)	5.91±3.48	6.13±3.78	6.17±4.06	5.88±3.38	5.39±2.61	5.92±3.22	6.04±3.46	6.07±3.66	5.98±3.76	5.48±3.38	5.09±2.57	5.63±3.18	6.00±3.66	6.25±3.77	6.51±4.00
Alpha-Tocopherol [c]	3.77±1.68	3.88±1.8	3.90±1.91	3.75±1.61	3.49±1.28	3.80±1.57	3.85±1.65	3.85±1.73	3.79±1.77	3.50±1.62	3.29±1.26	3.61±1.52	3.80±1.73	3.95±1.79	4.12±1.89
Total Alpha-Toc Eq [c]	4.99±3.39	5.24±3.71	5.35±4.00	5.14±3.32	4.80±2.55	5.03±3.14	5.18±3.39	5.25±3.59	5.21±3.7	4.84±3.32	4.53±2.52	4.94±3.13	5.20±3.61	5.35±3.71	5.49±3.94
Natural Alpha-Tocopherol [c]	3.34±0.85	3.39±0.81	3.39±0.80	3.35±0.74	3.23±0.69	3.41±0.83	3.42±0.78	3.39±0.76	3.33±0.75	3.15±0.75	3.05±0.68	3.25±0.73	3.35±0.75	3.46±0.81	3.59±0.83
Synthetic Alpha-Tocopherol [c]	0.95±3.28	1.08±3.63	1.12±3.93	0.88±3.23	0.58±2.42	0.85±3.02	0.95±3.29	1.02±3.50	1.01±3.63	0.78±3.22	0.55±2.4	0.79±3.05	1.02±3.53	1.10±3.63	1.17±3.86
Beta-Tocopherol [c]	0.16±0.06	0.17±0.06	0.17±0.06	0.17±0.06	0.17±0.06	0.16±0.05	0.17±0.06	0.17±0.06	0.17±0.06	0.17±0.06	0.16±0.06	0.16±0.06	0.17±0.06	0.17±0.06	0.18±0.06
Delta-Tocopherol [c]	1.08±0.65	1.22±0.70	1.34±0.74	1.48±0.8	1.62±0.83	1.21±0.73	1.30±0.76	1.36±0.77	1.40±0.78	1.45±0.79	1.52±0.79	1.45±0.78	1.36±0.77	1.26±0.75	1.14±0.70
Gamma-Tocopherol [c]	6.18±2.83	6.83±3.02	7.43±3.17	8.15±3.32	9.07±3.45	6.85±3.15	7.23±3.19	7.50±3.25	7.78±3.33	8.29±3.5	8.56±3.33	8.12±3.36	7.54±3.30	7.02±3.22	6.42±2.94
Vitamin K (NDS Value) [d]	63.79±48.38	63.92±47.55	62.51±45.4	58.82±38.12	50.14±29.00	65.93±48.96	63.56±44.19	61.38±43.63	58.85±40.51	49.45±32.17	43.56±26.59	52.79±36.88	59.26±39.86	67.20±48.43	76.37±49.48
Calcium [c]	577.47±216.8	560.32±210.54	527.97±203.07	483.65±183.89	409.75±155.73	533.08±205.09	530.72±197.51	525.13±201.29	511.22±207.48	459.01±200.88	419.10±167.34	470.06±182.72	507.76±196.14	548.51±204.03	613.72±212.58
Copper [c]	0.68±0.16	0.68±0.17	0.67±0.17	0.65±0.19	0.61±0.20	0.67±0.17	0.67±0.17	0.66±0.17	0.65±0.18	0.63±0.22	0.56±0.18	0.62±0.18	0.66±0.18	0.70±0.16	0.74±0.15
Iron [c]	7.97±2.47	8.17±2.62	8.19±2.75	7.92±2.43	7.31±2.04	7.73±2.30	7.91±2.41	8.01±2.54	8.07±2.62	7.84±2.57	7.07±2.11	7.59±2.35	7.99±2.53	8.29±2.54	8.62±2.61
Magnesium [c]	180.08±35.11	174.88±34.51	166.63±32.9	154.49±30.18	133.59±27.71	177.08±34.28	169.69±33.35	164.31±33.51	157.7±34.1	140.88±35.11	126.19±24.25	146.99±25.14	162.10±26.38	177.29±28.33	197.10±29.5
Manganese [c]	2.53±0.73	2.28±0.66	2.1±0.6	1.87±0.55	1.52±0.48	2.55±0.73	2.22±0.61	2.05±0.59	1.88±0.59	1.60±0.59	1.59±0.56	1.85±0.60	2.06±0.63	2.27±0.65	2.53±0.65
Phosphorous [c]	752.26±175.03	750.92±170.55	732.6±162.89	708.65±153.39	657.37±138.9	736.47±166.65	736.31±159.08	730.1±160.48	719.68±164.07	679.25±165.03	618.90±132.51	679.86±139.82	718.91±148.56	761.57±157.38	822.58±165.09
Potassium [c]	1859.89±426.88	1815.51±412.04	1727.93±387.83	1598.56±353.23	1383.99±319.35	1882.33±416.07	1780.93±382.86	1709.19±375.94	1616.39±376.7	1397.05±370.72	1296.40±295.03	1522.83±308.63	1688.10±335.03	1848.28±359.56	2030.27±360.14
Selenium [d]	53.88±10.75	55.71±10.88	56.51±10.94	57.83±11.36	57.96±12.49	55.49±11.21	56.33±10.83	56.48±10.87	56.84±11.26	56.74±12.69	53.21±11.43	55.52±11.16	56.58±11.01	57.63±11.29	58.95±11.28

Sodium [c]	1637.7±306.31	1673.95±292.14	1688.42±288.29	1707.1±283.09	1694.6±296.79	1686.07±309.87	1695.03±285.18	1688.04±284.25	1680.28±283.26	1652.35±306.4	1654.46±303.67	1682.87±294.43	1692.13±293.11	1699.65±293.78	1672.67±284.57
Zinc [c]	6.47±2.12	6.73±2.3	6.85±2.47	6.8±2.26	6.69±2.17	6.66±2.09	6.77±2.17	6.81±2.29	6.78±2.36	6.54±2.41	5.91±1.91	6.47±2.09	6.81±2.31	7.05±2.35	7.31±2.39
**Bioactives**															
Total carotenoids	7810.54 ± 4273.75	7807.54 ± 4294.25	7495.03 ± 4171.15	6853.16 ± 3667.98	5549.34 ± 2995.46	7901.67 ± 4341.41	7622.46 ± 4022.16	7313.57 ± 3940.93	6931.63 ± 3844.56	5746.31 ± 3463.92	4862.60 ± 2745.43	6202.39 ± 3437.90	7214.05 ± 3788.73	8199.10 ± 4284.53	9037.48 ± 4159.67
Alpha-Carotene [d]	529.42±423.38	523.23±413.15	489.69±405.16	429.74±336.98	332.46±253.77	578.68±474.36	522.37±391.75	473.46±359.21	418.7±326.04	311.34±251.32	276.98±271.69	382.32±305.58	461.76±350.47	547.85±402.15	635.62±434.86
Beta-Carotene [d]	2461.44±1683.56	2420.89±1612.9	2275.49±1547.43	2016.33±1300.09	1563.77±1018.52	2525.71±1765.48	2356.92±1514.15	2220.56±1468.7	2043.55±1350.36	1591.19±1096.82	1289.21±928.21	1783.70±1216.51	2153.74±1374.41	2548.49±1577.92	2962.78±1640.36
Beta-Cryptoxanthin [d]	110.8±68.25	112.46±69.99	105.94±66.86	91.76±58.43	70.03±45.62	104.49±65.87	105.92±65.49	103.3±65.5	98.47±65.12	78.82±55.87	57.88±45.31	83.19±54.13	101.32±61.77	116.61±65.79	131.98±65.67
Lycopene [d]	3374.85±2040.42	3437.29±2129.94	3363.12±2127.97	3181.78±2032.62	2713.38±1761.83	3354.82±1947.2	3358.21±1971.65	3291.62±2001.81	3210.51±2115.97	2855.27±2115.31	2498.71±1699.81	2973.78±1903.67	3328.50±2049.2	3599.11±2193.25	3670.33±2086.04
Lutein+Zeaxanthin [d]	1334.04±1252.67	1313.65±1246.34	1260.79±1191.6	1133.56±988.33	869.71±728.4	1337.97±1275.73	1279.04±1155.27	1224.63±1144.83	1160.4±1057.13	909.7±834.19	739.81±667.81	979.39±954.86	1168.73±1041.86	1387.04±1267.53	1636.77±1288.75
Oxalic Acid [c]	230.37±98.19	224.85±95.22	213.01±90.73	192.46±78.03	156.33±62.58	231.55±99.18	219.22±88.83	208.65±86.97	195.48±83.8	162.11±73.55	141.62±56.76	177.75±71.65	204.75±81.59	232.32±91.79	260.57±92.41
Phytic Acid [c]	392.52±135.8	382.96±125.86	364.87±119.28	335.92±109.76	287.5±95.31	370.57±123.36	366.54±119.94	358.99±120.95	348.65±122.02	319±126.48	273.86±93.52	318.84±102.88	350.54±110.07	384.39±116.26	436.13±128.56
**Isoflavones[c]**															
Total isoflavones	1.44 ± 3.87	1.15 ± 3.26	0.94 ± 2.63	0.76 ± 2.20	0.55 ± 1.42	1.09 ± 2.53	0.99 ± 2.66	0.95 ± 2.91	0.90 ± 2.73	0.91 ± 3.23	0.59 ± 1.19	0.72 ± 1.78	0.84 ± 2.21	1.07 ± 2.88	1.62 ± 4.63
Biochanin A	0.01±0.01	0.01±0.01	0.01±0.01	0.01±0.01	0.00±0.01	0.01±0.01	0.01±0.01	0.01±0.01	0.01±0.01	0.00±0.01	0.00±0.01	0.00±0.01	0.01±0.01	0.01±0.01	0.01±0.01
Coumestrol	0.06±0.04	0.05±0.04	0.04±0.03	0.04±0.03	0.03±0.02	0.07±0.05	0.05±0.03	0.04±0.02	0.03±0.02	0.02±0.01	0.04±0.04	0.04±0.04	0.04±0.04	0.04±0.03	0.04±0.03
Daidzein	0.60±1.46	0.48±1.23	0.39±0.98	0.32±0.82	0.23±0.53	0.48±0.96	0.42±1.01	0.40±1.1	0.36±1.02	0.35±1.2	0.26±0.45	0.31±0.66	0.36±0.82	0.44±1.07	0.65±1.76
Formononetin	0.00±0.01	0.00±0.00	0.00±0.00	0.00±0.00	0.00±0.00	0.00±0.00	0.00±0.00	0.00±0.00	0.00±0.00	0.00±0.00	0.00±0.00	0.00±0.00	0.00±0.00	0.00±0.00	0.00±0.01
Genistein	0.71±2.16	0.56±1.82	0.45±1.48	0.36±1.23	0.26±0.79	0.48±1.41	0.46±1.48	0.46±1.62	0.45±1.52	0.48±1.81	0.27±0.66	0.34±1.00	0.40±1.24	0.52±1.61	0.82±2.58
Glycitein	0.07±0.25	0.05±0.22	0.04±0.18	0.03±0.15	0.02±0.1	0.04±0.17	0.04±0.17	0.05±0.19	0.05±0.18	0.05±0.22	0.02±0.08	0.03±0.13	0.04±0.15	0.05±0.20	0.09±0.30
Ash	10.39±1.73	10.31±1.66	10.05±1.58	9.69±1.50	9.02±1.45	10.63±1.75	10.25±1.53	9.99±1.49	9.66±1.48	8.94±1.54	8.82±1.46	9.47±1.45	9.93±1.49	10.39±1.56	10.86±1.55

a.EDIH, empirical dietary index for hyperinsulinemia score assessing the ability of the dietary pattern to contribute to insulin hypersecretion - higher EDIH scores reflect more hyperinsulinemic dietary patterns;
EDIP, empirical dietary inflammatory pattern score assessing the ability of the dietary pattern to contribute to chronic systemic inflammation - higher EDIP scores reflect more pro-inflammatory dietary patterns;

HEI-2015, healthy eating index-2015 assessing adherence to the 2015-2020 Dietary Guidelines for Americans - higher HEI-2015 scores are indicative of greater adherence and higher dietary quality. EDIH and EDIP are positively correlated, whereas both scores are inversely correlated with HEI-2015, i.e., more hyperinsulinemic or pro-inflamamtory dietary patterns are of lower dietary quality. Each dietary score was adjusted for total energy intake using the residual method. All nutrients listed are from dietary sources.

b. nutrients were g/d/1000kcal

c. nutrients were mg/d/1000kcal

d. nutrients were mcg/d/1000kcal

Supplementary Table S5. Hazard ratios (95% CI) for the associations of dietary patterns with total and site-specific cancers, further adjusted for body mass index and type 2 diabetes[a]

Dietary pattern	Cancer site[b]/statistical model	Quintile 1	Quintile 2	Quintile 3	Quintile 4	Quintile 5	P for linear trend[c]	1-SD increment in dietary score	P for continuous dietary score[d]
	**Total cancer (except non-melanoma skin cancer)**								
	**Cases/non cases**	3934/18559	3861/18633	3772/18722	3594/18900	3607/18886			
EDIH	MV BMI-adjusted	1	**1.04 (0.99, 1.09)**	**1.06 (1.01, 1.11)**	**1.03 (0.98, 1.08)**	**1.07 (1.02, 1.12)**	0.0216	**1.02 (1.00, 1.03)**	0.0499
EDIH	MV DIAB-adjusted	1	**1.04 (1.00, 1.09)**	**1.06 (1.01, 1.11)**	**1.04 (1.00, 1.10)**	**1.09 (1.04, 1.15)**	0.0011	**1.02 (1.01, 1.04)**	0.0032
	**Cases/non cases**	4131/18362	4027/18467	3703/18791	3608/18886	3299/19194			
EDIP	MV BMI-adjusted	1	**1.06 (1.01, 1.11)**	**1.02 (0.97, 1.07)**	**1.06 (1.00, 1.12)**	**1.06 (1.00, 1.12)**	0.0786	**1.03 (1.01, 1.05)**	0.01
EDIP	MV DIAB-adjusted	1	**1.06 (1.01, 1.11)**	**1.02 (0.97, 1.08)**	**1.07 (1.01, 1.13)**	**1.08 (1.02, 1.14)**	0.0188	**1.03 (1.01, 1.06)**	0.0014
	**Cases/non cases**	3697/18796	3740/18753	3786/18708	3750/18743	3795/18698			
HEI-2015	MV BMI-adjusted	1	0.98 (0.94, 1.03)	0.97 (0.93, 1.02)	**0.95 (0.90, 0.99)**	**0.95 (0.90, 0.99)**	0.0086	**0.97 (0.96, 0.99)**	0.0006
HEI-2015	MV DIAB-adjusted	1	0.98 (0.93, 1.02)	0.97 (0.92, 1.01)	**0.94 (0.89, 0.98)**	**0.93 (0.89, 0.98)**	0.0006	**0.97 (0.95, 0.98)**	<.0001
	**Colorectal cancer**								
	**Cases/non cases**	301/21146	309/21139	372/21075	306/21142	320/21127			
EDIH	MV BMI-adjusted	1	1.06 (0.89, 1.24)	1.29 (1.10, 1.52)	1.05 (0.88, 1.25)	1.14 (0.95, 1.36)	0.2100	**1.05 (0.99, 1.11)**	0.1084
EDIH	MV DIAB-adjusted	1	1.05 (0.89, 1.24)	1.30 (1.10, 1.53)	1.07 (0.90, 1.28)	**1.19 (1.00, 1.42)**	0.0698	**1.06 (1.00, 1.12)**	0.0348
	**Cases/non cases**	315/21132	333/21115	338/21109	319/21129	303/21144			
EDIP	MV BMI-adjusted	1	1.13 (0.95, 1.34)	1.17 (0.98, 1.41)	1.17 (0.96, 1.42)	1.18 (0.96, 1.46)	0.1295	**1.07 (0.99, 1.15)**	0.0891
EDIP	MV DIAB-adjusted	1	**1.15 (0.97, 1.36)**	1.21 (1.01, 1.45)	1.21 (0.99, 1.47)	**1.25 (1.01, 1.54)**	0.0437	**1.08 (1.01, 1.17)**	0.0304
	**Cases/non cases**	342/21105	337/21110	316/21131	297/21150	316/21131			
HEI-2015	MV BMI-adjusted	1	0.97 (0.83, 1.13)	0.92 (0.78, 1.08)	**0.84 (0.72, 1.00)**	**0.88 (0.75, 1.04)**	0.0468	**0.94 (0.89, 0.99)**	0.0271

HEI-2015	MV DIAB-adjusted	1	0.95 (0.82, 1.11)	0.91 (0.78, 1.06)	**0.83 (0.70, 0.97)**	**0.85 (0.72, 1.00)**	0.0153	**0.93 (0.88, 0.98)**	0.007
	Colon cancer								
	**Cases/non cases**	250/21217	264/21203	312/21155	254/21213	266/21201			
EDIH	MV BMI-adjusted	1	1.09 (0.91, 1.30)	1.32 (1.10, 1.58)	1.06 (0.88, 1.29)	1.16 (0.95, 1.41)	0.2156	**1.06 (0.99, 1.12)**	0.0755
EDIH	MV DIAB-adjusted	1	1.08 (0.90, 1.30)	1.32 (1.11, 1.59)	1.08 (0.89, 1.31)	**1.21 (1.00, 1.47)**	0.0829	**1.07 (1.01, 1.14)**	0.0259
	**Cases/non cases**	260/21207	286/21181	279/21188	266/21201	255/21212			
EDIP	MV BMI-adjusted	1	1.18 (0.98, 1.42)	1.18 (0.97, 1.44)	1.20 (0.96, 1.48)	**1.23 (0.98, 1.55)**	0.1022	**1.08 (1.00, 1.17)**	0.0569
EDIP	MV DIAB-adjusted	1	**1.19 (0.99, 1.44)**	**1.21 (0.99, 1.48)**	**1.23 (0.99, 1.53)**	**1.30 (1.03, 1.64)**	0.0394	**1.10 (1.02, 1.20)**	0.02
	**Cases/non cases**	285/21181	284/21183	264/21203	249/21218	264/21202			
HEI-2015	MV BMI-adjusted	1	0.97 (0.82, 1.15)	0.91 (0.77, 1.08)	**0.83 (0.70, 0.99)**	**0.86 (0.72, 1.03)**	0.03	**0.93 (0.88, 0.99)**	0.0203
HEI-2015	MV DIAB-adjusted	1	0.95 (0.80, 1.13)	0.90 (0.76, 1.07)	**0.81 (0.67, 0.96)**	**0.82 (0.69, 0.99)**	0.0085	**0.92 (0.87, 0.98)**	0.0047
	Proximal colon cancer								
	**Cases/non cases**	141/21362	160/21344	204/21299	155/21349	168/21335			
EDIH	MV BMI-adjusted	1	1.16 (0.92, 1.47)	1.53 (1.22, 1.93)	1.16 (0.91, 1.49)	**1.36 (1.05, 1.75)**	0.0359	**1.10 (1.02, 1.18)**	0.0161
EDIH	MV DIAB-adjusted	1	1.16 (0.92, 1.48)	1.55 (1.23, 1.96)	1.18 (0.92, 1.52)	**1.41 (1.10, 1.82)**	0.0142	**1.11 (1.03, 1.20)**	0.0074
	**Cases/non cases**	147/21356	185/21319	178/21325	160/21344	158/21345			
EDIP	MV BMI-adjusted	1	1.35 (1.06, 1.72)	1.32 (1.02, 1.71)	1.27 (0.96, 1.68)	**1.37 (1.01, 1.85)**	0.1001	**1.08 (0.97, 1.20)**	0.1476
EDIP	MV DIAB-adjusted	1	1.38 (1.08, 1.75)	1.36 (1.05, 1.76)	1.30 (0.98, 1.72)	**1.42 (1.05, 1.92)**	0.0636	**1.09 (0.98, 1.21)**	0.0996
	**Cases/non cases**	173/21330	176/21327	169/21334	151/21352	159/21344			
HEI-2015	MV BMI-adjusted	1	0.97 (0.78, 1.20)	0.93 (0.74, 1.15)	**0.80 (0.63, 1.00)**	**0.82 (0.65, 1.04)**	0.0323	**0.94 (0.87, 1.01)**	0.0858
HEI-2015	MV DIAB-adjusted	1	0.94 (0.76, 1.17)	0.92 (0.74, 1.14)	**0.78 (0.62, 0.98)**	**0.80 (0.63, 1.00)**	0.0172	**0.93 (0.86, 1.00)**	0.0428
	Distal colon and rectal cancer								
	**Cases/non cases**	127/21406	116/21418	132/21401	119/21415	133/21400			
EDIH	MV BMI-adjusted	1	0.90 (0.69, 1.18)	0.95 (0.73, 1.25)	0.91 (0.69, 1.20)	1.00 (0.76, 1.33)	0.9318	1.01 (0.92, 1.10)	0.8816
EDIH	MV DIAB-adjusted	1	0.90 (0.69, 1.18)	1.00 (0.76, 1.31)	0.95 (0.72, 1.26)	1.09 (0.82, 1.45)	0.4564	1.03 (0.94, 1.13)	0.4677
	**Cases/non cases**	141/21392	109/21425	119/21414	131/21403	127/21406			

EDIP	MV BMI-adjusted	1	0.79 (0.60, 1.05)	0.86 (0.64, 1.14)	1.00 (0.73, 1.35)	0.97 (0.70, 1.36)	0.7688	1.07 (0.95, 1.21)	0.2646
EDIP	MV DIAB-adjusted	1	0.81 (0.62, 1.08)	0.91 (0.68, 1.22)	1.03 (0.76, 1.41)	1.06 (0.76, 1.47)	0.4586	**1.10 (0.98, 1.24)**	0.1137
	**Cases/non cases**	136/21397	138/21395	114/21419	117/21416	122/21411			
HEI-2015	MV BMI-adjusted	1	1.03 (0.80, 1.33)	0.91 (0.70, 1.18)	0.89 (0.69, 1.17)	0.97 (0.74, 1.27)	0.5346	0.96 (0.88, 1.05)	0.3734
HEI-2015	MV DIAB-adjusted	1	1.02 (0.79, 1.32)	0.88 (0.67, 1.14)	0.87 (0.67, 1.14)	0.91 (0.69, 1.19)	0.2688	**0.94 (0.86, 1.03)**	0.166
	Intestinal polyps								
	**Cases/non cases**	1991/18700	2030/18662	2134/18558	2211/18481	2308/18383			
EDIH	MV BMI-adjusted	1	**1.05 (0.98, 1.12)**	**1.13 (1.06, 1.21)**	**1.16 (1.09, 1.24)**	**1.18 (1.10, 1.27)**	<.0001	**1.06 (1.04, 1.08)**	<.0001
EDIH	MV DIAB-adjusted	1	**1.05 (0.98, 1.12)**	**1.14 (1.07, 1.22)**	**1.18 (1.11, 1.27)**	**1.22 (1.14, 1.31)**	<.0001	**1.07 (1.05, 1.09)**	<.0001
	**Cases/non cases**	2143/18548	2144/18548	2113/18579	2081/18611	2193/18498			
EDIP	MV BMI-adjusted	1	**1.05 (0.98, 1.12)**	**1.06 (0.99, 1.14)**	**1.07 (0.99, 1.15)**	**1.13 (1.04, 1.22)**	0.0055	**1.05 (1.02, 1.08)**	0.0011
EDIP	MV DIAB-adjusted	1	**1.05 (0.98, 1.12)**	**1.08 (1.00, 1.16)**	**1.08 (1.00, 1.17)**	**1.16 (1.07, 1.26)**	0.0006	**1.06 (1.03, 1.09)**	<.0001
	**Cases/non cases**	2307/18384	2223/18468	2172/18520	2075/18616	1896/18795			
HEI-2015	MV BMI-adjusted	1	0.98 (0.92, 1.04)	0.96 (0.90, 1.02)	**0.91 (0.86, 0.97)**	**0.86 (0.80, 0.92)**	<.0001	**0.95 (0.93, 0.97)**	<.0001
HEI-2015	MV DIAB-adjusted	1	0.96 (0.91, 1.02)	**0.94 (0.88, 1.00)**	**0.89 (0.84, 0.95)**	**0.84 (0.78, 0.89)**	<.0001	**0.94 (0.92, 0.96)**	<.0001
	**Invasive Breast cancer**								
	**Cases/non cases**	899/17565	921/17544	827/17637	843/17622	903/17561			
EDIH	MV BMI-adjusted	1	1.08 (0.98, 1.19)	1.00 (0.91, 1.11)	1.04 (0.94, 1.15)	**1.14 (1.02, 1.26)**	0.0458	**1.03 (0.99, 1.06)**	0.1074
EDIH	MV DIAB-adjusted	1	1.09 (0.99, 1.20)	1.02 (0.92, 1.12)	1.06 (0.96, 1.18)	**1.19 (1.07, 1.32)**	0.0034	**1.04 (1.01, 1.08)**	0.0103
	**Cases/non cases**	926/17538	936/17529	874/17590	866/17599	791/17673			
EDIP	MV BMI-adjusted	1	1.09 (0.98, 1.20)	1.06 (0.95, 1.18)	1.11 (0.99, 1.25)	1.09 (0.96, 1.23)	0.1785	**1.04 (0.99, 1.08)**	0.1053
EDIP	MV DIAB-adjusted	1	1.09 (0.99, 1.20)	1.07 (0.96, 1.19)	**1.13 (1.01, 1.27)**	**1.12 (0.99, 1.27)**	0.058	**1.05 (1.00, 1.10)**	0.0281
	**Cases/non cases**	872/17592	867/17597	847/17617	869/17595	938/17526			
HEI-2015	MV BMI-adjusted	1	0.96 (0.87, 1.05)	0.92 (0.84, 1.01)	0.94 (0.85, 1.04)	1.02 (0.92, 1.13)	0.8739	1.00 (0.97, 1.04)	0.8875
HEI-2015	MV DIAB-adjusted	1	0.95 (0.86, 1.04)	0.90 (0.82, 1.00)	0.92 (0.83, 1.01)	0.99 (0.90, 1.09)	0.6071	0.99 (0.96, 1.02)	0.5792

	ER+								
	**Cases/non cases**	745/17792	747/17790	685/17852	688/17849	714/17823			
EDIH	MV BMI-adjusted	1	1.07 (0.97, 1.19)	1.02 (0.92, 1.14)	1.04 (0.93, 1.17)	**1.10 (0.98, 1.24)**	0.1639	1.01 (0.98, 1.05)	0.4995
EDIH	MV DIAB-adjusted	1	1.08 (0.97, 1.20)	1.04 (0.93, 1.16)	1.07 (0.96, 1.20)	**1.16 (1.04, 1.31)**	0.0164	**1.03 (1.00, 1.07)**	0.089
	**Cases/non cases**	769/17768	768/17769	726/17811	704/17833	612/17925			
EDIP	MV BMI-adjusted	1	1.08 (0.97, 1.21)	1.08 (0.96, 1.21)	1.11 (0.98, 1.26)	1.04 (0.90, 1.19)	0.4851	1.02 (0.97, 1.06)	0.5082
EDIP	MV DIAB-adjusted	1	1.09 (0.98, 1.22)	1.09 (0.97, 1.23)	1.14 (1.00, 1.29)	1.08 (0.94, 1.24)	0.1944	**1.03 (0.98, 1.08)**	0.2051
	**Cases/non cases**	694/17842	692/17845	695/17841	720/17817	778/17758			
HEI-2015	MV BMI-adjusted	1	0.96 (0.86, 1.06)	0.95 (0.85, 1.06)	0.98 (0.88, 1.09)	1.06 (0.95, 1.19)	0.2687	1.02 (0.98, 1.06)	0.3057
HEI-2015	MV DIAB-adjusted	1	0.95 (0.85, 1.05)	0.93 (0.84, 1.04)	0.95 (0.86, 1.06)	1.03 (0.92, 1.14)	0.6788	1.01 (0.97, 1.04)	0.7569
	ER-								
	**Cases/non cases**	100/18600	123/18577	105/18595	100/18600	127/18573			
EDIH	MV BMI-adjusted	1	1.22 (0.93, 1.60)	1.05 (0.79, 1.41)	1.02 (0.76, 1.38)	**1.34 (1.00, 1.80)**	0.1455	**1.12 (1.02, 1.22)**	0.0198
EDIH	MV DIAB-adjusted	1	1.22 (0.93, 1.60)	1.05 (0.79, 1.40)	1.01 (0.75, 1.36)	**1.30 (0.97, 1.75)**	0.2063	**1.10 (1.01, 1.21)**	0.0344
	**Cases/non cases**	108/18592	104/18596	106/18594	124/18576	113/18587			
EDIP	MV BMI-adjusted	1	0.98 (0.74, 1.31)	1.02 (0.75, 1.40)	1.24 (0.90, 1.71)	1.20 (0.85, 1.71)	0.1575	**1.12 (0.99, 1.26)**	0.0719
EDIP	MV DIAB-adjusted	1	0.98 (0.73, 1.31)	1.02 (0.75, 1.39)	1.23 (0.89, 1.70)	1.18 (0.83, 1.67)	0.1918	**1.11 (0.98, 1.25)**	0.0909
	**Cases/non cases**	123/18576	110/18590	107/18592	104/18596	111/18588			
HEI-2015	MV BMI-adjusted	1	0.86 (0.67, 1.12)	0.82 (0.63, 1.07)	0.79 (0.60, 1.03)	0.84 (0.64, 1.11)	0.1468	0.95 (0.87, 1.04)	0.298
HEI-2015	MV DIAB-adjusted	1	0.86 (0.67, 1.12)	0.82 (0.63, 1.07)	0.79 (0.60, 1.04)	0.85 (0.64, 1.11)	0.1652	0.96 (0.87, 1.05)	0.3257
	PR+								
	**Cases/non cases**	626/17945	636/17935	588/17983	575/17996	604/17967			
EDIH	MV BMI-adjusted	1	1.10 (0.98, 1.23)	1.06 (0.94, 1.20)	1.05 (0.93, 1.19)	**1.13 (1.00, 1.28)**	0.126	**1.02 (0.98, 1.06)**	0.3071
EDIH	MV DIAB-adjusted	1	1.11 (0.99, 1.24)	1.08 (0.96, 1.22)	1.09 (0.96, 1.23)	**1.20 (1.06, 1.36)**	0.008	**1.04 (1.00, 1.08)**	0.0309
	**Cases/non cases**	648/17923	649/17922	635/17936	597/17974	500/18071			
EDIP	MV BMI-adjusted	1	1.11 (0.99, 1.25)	1.15 (1.02, 1.31)	1.16 (1.01, 1.32)	1.04 (0.89, 1.21)	0.4215	1.02 (0.96, 1.07)	0.5488
EDIP	MV DIAB-adjusted	1	1.12 (1.00, 1.26)	1.17 (1.03, 1.33)	1.18 (1.03, 1.36)	1.09 (0.94, 1.27)	0.1407	**1.03 (0.98, 1.09)**	0.1983

	**Cases/non cases**	597/17973	580/17991	593/17977	615/17956	644/17926			
HEI-2015	MV BMI-adjusted	1	0.93 (0.83, 1.05)	0.94 (0.84, 1.06)	0.97 (0.87, 1.09)	1.02 (0.91, 1.15)	0.6067	1.01 (0.97, 1.05)	0.6053
HEI-2015	MV DIAB-adjusted	1	0.92 (0.82, 1.04)	0.92 (0.82, 1.03)	0.94 (0.84, 1.06)	0.98 (0.87, 1.10)	0.7878	0.99 (0.96, 1.03)	0.7714
	PR-								
	**Cases/non cases**	206/18464	222/18448	195/18475	204/18466	225/18445			
EDIH	MV BMI-adjusted	1	1.08 (0.89, 1.31)	0.97 (0.79, 1.19)	1.03 (0.83, 1.27)	1.17 (0.95, 1.45)	0.2193	**1.05 (0.98, 1.13)**	0.1383
EDIH	MV DIAB-adjusted	1	1.08 (0.89, 1.31)	0.96 (0.78, 1.19)	1.02 (0.83, 1.26)	1.16 (0.94, 1.43)	0.2618	**1.05 (0.98, 1.12)**	0.1711
	**Cases/non cases**	220/18450	214/18456	182/18488	222/18448	214/18456			
EDIP	MV BMI-adjusted	1	0.96 (0.79, 1.18)	0.83 (0.66, 1.04)	1.04 (0.83, 1.32)	1.07 (0.83, 1.37)	0.5163	**1.06 (0.97, 1.16)**	0.201
EDIP	MV DIAB-adjusted	1	0.96 (0.79, 1.18)	0.83 (0.66, 1.04)	1.04 (0.83, 1.31)	1.06 (0.82, 1.36)	0.5537	**1.06 (0.97, 1.16)**	0.2208
	**Cases/non cases**	212/18457	212/18458	204/18465	199/18471	225/18444			
HEI-2015	MV BMI-adjusted	1	0.96 (0.79, 1.16)	0.91 (0.74, 1.10)	0.87 (0.72, 1.07)	0.98 (0.80, 1.20)	0.5911	0.99 (0.93, 1.06)	0.7876
HEI-2015	MV DIAB-adjusted	1	0.96 (0.79, 1.16)	0.90 (0.74, 1.10)	0.87 (0.72, 1.07)	0.98 (0.81, 1.20)	0.5991	0.99 (0.93, 1.06)	0.7932
	HER2+								
	**Cases/non cases**	86/18628	85/18629	86/18628	85/18629	100/18614			
EDIH	MV BMI-adjusted	1	0.99 (0.73, 1.35)	1.02 (0.74, 1.40)	1.01 (0.73, 1.40)	1.21 (0.87, 1.68)	0.2444	**1.08 (0.97, 1.19)**	0.1506
EDIH	MV DIAB-adjusted	1	0.99 (0.73, 1.35)	1.03 (0.75, 1.41)	1.02 (0.74, 1.42)	1.23 (0.89, 1.70)	0.1898	**1.09 (0.98, 1.20)**	0.1113
	**Cases/non cases**	81/18633	97/18617	91/18623	86/18628	87/18627			
EDIP	MV BMI-adjusted	1	1.25 (0.91, 1.72)	1.20 (0.85, 1.70)	1.19 (0.82, 1.73)	1.26 (0.85, 1.88)	0.3513	1.08 (0.95, 1.24)	0.2447
EDIP	MV DIAB-adjusted	1	1.25 (0.91, 1.72)	1.21 (0.86, 1.71)	1.20 (0.83, 1.74)	1.28 (0.86, 1.91)	0.3075	1.09 (0.95, 1.25)	0.2062
	**Cases/non cases**	97/18616	84/18630	84/18629	90/18624	87/18626			
HEI-2015	MV BMI-adjusted	1	0.85 (0.63, 1.14)	0.85 (0.63, 1.15)	0.92 (0.68, 1.24)	0.91 (0.67, 1.24)	0.6721	0.98 (0.88, 1.08)	0.6887
HEI-2015	MV DIAB-adjusted	1	0.84 (0.63, 1.13)	0.84 (0.62, 1.13)	0.91 (0.68, 1.22)	0.89 (0.66, 1.22)	0.5789	0.97 (0.88, 1.08)	0.5902
	HER2-								
	**Cases/non cases**	698/17957	708/17948	639/18016	647/18009	660/17995			
EDIH	MV BMI-adjusted	1	1.09 (0.98, 1.21)	1.02 (0.91, 1.14)	1.05 (0.94, 1.18)	**1.10 (0.97, 1.24)**	0.2238	1.02 (0.98, 1.06)	0.3173
EDIH	MV DIAB-adjusted	1	1.09 (0.98, 1.22)	1.03 (0.92, 1.16)	1.08 (0.96, 1.21)	**1.15 (1.02, 1.29)**	0.0432	**1.04 (1.00, 1.07)**	0.0686

	**Cases/non cases**	726/17929	722/17934	665/17990	660/17996	579/18076			
EDIP	MV BMI-adjusted	1	1.09 (0.97, 1.22)	1.05 (0.93, 1.19)	1.11 (0.98, 1.27)	1.05 (0.91, 1.22)	0.4033	1.02 (0.98, 1.08)	0.3273
EDIP	MV DIAB-adjusted	1	1.09 (0.98, 1.22)	1.06 (0.94, 1.20)	1.13 (0.99, 1.29)	1.09 (0.95, 1.26)	0.1887	**1.04 (0.99, 1.09)**	0.1405
	**Cases/non cases**	654/18000	652/18003	662/17993	657/17998	727/17928			
HEI-2015	MV BMI-adjusted	1	0.96 (0.86, 1.06)	0.95 (0.86, 1.06)	0.94 (0.84, 1.05)	1.04 (0.93, 1.17)	0.6159	1.01 (0.97, 1.05)	0.6315
HEI-2015	MV DIAB-adjusted	1	0.95 (0.85, 1.06)	0.94 (0.84, 1.05)	0.92 (0.82, 1.03)	1.01 (0.90, 1.13)	0.9456	1.00 (0.96, 1.04)	0.9115
	ER- PR- HER2+								
	**Cases/non cases**	25/18707	23/18710	25/18707	25/18708	22/18710			
EDIH	MV BMI-adjusted	1	0.92 (0.52, 1.65)	1.02 (0.57, 1.84)	1.03 (0.56, 1.88)	0.92 (0.49, 1.75)	0.9036	1.00 (0.82, 1.22)	0.9904
EDIH	MV DIAB-adjusted	1	0.92 (0.51, 1.65)	1.01 (0.56, 1.82)	1.01 (0.55, 1.82)	0.88 (0.47, 1.67)	0.7931	0.98 (0.81, 1.20)	0.8749
	**Cases/non cases**	19/18713	31/18702	21/18711	25/18708	24/18708			
EDIP	MV BMI-adjusted	1	1.89 (1.01, 3.53)	1.37 (0.67, 2.80)	1.73 (0.82, 3.64)	1.79 (0.80, 3.97)	0.2604	1.06 (0.82, 1.38)	0.6553
EDIP	MV DIAB-adjusted	1	1.88 (1.00,3.51)	1.35 (0.66, 2.77)	1.70 (0.81, 3.58)	1.73 (0.78, 3.83)	0.2996	1.05(0.81, 1.36)	0.7207
	**Cases/non cases**	30/18701	22/18710	23/18709	23/18709	22/18710			
HEI-2015	MV BMI-adjusted	1	0.72 (0.41, 1.25)	0.74 (0.43, 1.29)	0.75 (0.43, 1.32)	0.74 (0.41, 1.32)	0.3243	0.92 (0.76, 1.12)	0.4273
HEI-2015	MV DIAB-adjusted	1	0.72 (0.42, 1.26)	0.75 (0.43, 1.31)	0.76 (0.44, 1.34)	0.76 (0.42, 1.36)	0.3731	0.93 (0.77, 1.13)	0.4854
	Luminal A								
	**Cases/non cases**	640/18026	625/18041	575/18091	582/18084	576/18090			
EDIH	MV BMI-adjusted	1	1.06 (0.94, 1.18)	1.01 (0.90, 1.14)	1.05 (0.93, 1.18)	1.06 (0.93, 1.20)	0.4648	1.00 (0.96, 1.04)	0.8666
EDIH	MV DIAB-adjusted	1	1.06 (0.95, 1.19)	1.03 (0.91, 1.16)	1.08 (0.96, 1.22)	**1.11 (0.98, 1.26)**	0.1034	1.02 (0.98, 1.06)	0.2814
	**Cases/non cases**	660/18006	655/18011	599/18067	577/18089	507/18159			
EDIP	MV BMI-adjusted	1	1.09 (0.97, 1.23)	1.06 (0.93, 1.20)	1.09 (0.95, 1.25)	1.04 (0.89, 1.20)	0.6258	1.01 (0.96, 1.07)	0.5888
EDIP	MV DIAB-adjusted	1	1.10 (0.98, 1.24)	1.07 (0.94, 1.22)	1.11 (0.97, 1.28)	1.08 (0.93, 1.26)	0.3073	1.03 (0.98, 1.08)	0.2782
	**Cases/non cases**	579/18086	582/18084	593/18072	593/18073	651/18014			
HEI-2015	MV BMI-adjusted	1	0.96 (0.86, 1.08)	0.96 (0.86, 1.08)	0.96 (0.85, 1.08)	1.05 (0.94, 1.19)	0.4815	1.01 (0.97, 1.05)	0.5700
HEI-2015	MV DIAB-adjusted	1	0.95 (0.85, 1.07)	0.95 (0.84, 1.06)	0.93 (0.83, 1.05)	1.02 (0.90, 1.14)	0.9266	1.00 (0.96, 1.04)	0.9482
	Luminal B								

	**Cases/non cases**	59/18662	61/18660	60/18662	59/18662	76/18645			
EDIH	MV BMI-adjusted	1	1.04 (0.72, 1.51)	1.05 (0.72, 1.53)	1.04 (0.70, 1.52)	1.35 (0.92, 1.98)	0.1418	**1.11 (0.99, 1.25)**	0.0827
EDIH	MV DIAB-adjusted	1	1.05 (0.72, 1.51)	1.06 (0.72, 1.54)	1.05 (0.71, 1.54)	**1.39 (0.95, 2.04)**	0.0927	**1.13 (1.00, 1.27)**	0.0497
	**Cases/non cases**	61/18660	65/18656	69/18653	59/18662	61/18660			
EDIP	MV BMI-adjusted	1	1.06 (0.73, 1.55)	1.14 (0.77, 1.69)	1.01 (0.65, 1.56)	1.08 (0.68, 1.71)	0.8397	1.08 (0.92, 1.27)	0.3545
EDIP	MV DIAB-adjusted	1	1.07 (0.73, 1.56)	1.15 (0.77, 1.71)	1.02 (0.66, 1.58)	1.11 (0.70, 1.76)	0.742	1.09 (0.93, 1.28)	0.2858
	**Cases/non cases**	65/18655	59/18662	60/18661	67/18654	64/18656			
HEI-2015	MV BMI-adjusted	1	0.89 (0.62, 1.27)	0.91 (0.64, 1.31)	1.03 (0.72, 1.47)	1.01 (0.70, 1.46)	0.7698	1.02 (0.90, 1.15)	0.7911
HEI-2015	MV DIAB-adjusted	1	0.88 (0.62, 1.26)	0.90 (0.63, 1.28)	1.01 (0.71, 1.44)	0.97 (0.68, 1.41)	0.9114	1.00 (0.89, 1.13)	0.9397
	Triple negative								
	**Cases/non cases**	57/18672	83/18647	61/18668	63/18667	84/18645			
EDIH	MV BMI-adjusted	1	1.43 (1.01, 2.02)	1.06 (0.72, 1.54)	1.10 (0.74, 1.62)	**1.52 (1.04, 2.21)**	0.1159	**1.16 (1.04, 1.31)**	0.0086
EDIH	MV DIAB-adjusted	1	1.43 (1.01, 2.02)	1.05 (0.72, 1.54)	1.09 (0.74, 1.60)	**1.48 (1.01, 2.15)**	0.1523	**1.15 (1.03, 1.29)**	0.0138
	**Cases/non cases**	66/18663	65/18665	65/18664	81/18649	7/18658			
EDIP	MV BMI-adjusted	1	0.97 (0.67, 1.40)	0.98 (0.66, 1.45)	1.24 (0.83, 1.86)	1.14 (0.73, 1.78)	0.3461	1.11 (0.95, 1.30)	0.1842
EDIP	MV DIAB-adjusted	1	0.97 (0.67, 1.40)	0.98 (0.66, 1.45)	1.23 (0.82, 1.85)	1.12 (0.72, 1.75)	0.3865	1.10 (0.95, 1.29)	0.2103
	**Cases/non cases**	75/18653	70/18659	69/18660	61/18668	73/18656			
HEI-2015	MV BMI-adjusted	1	0.91 (0.66, 1.27)	0.89 (0.64, 1.24)	0.77 (0.54, 1.09)	0.92 (0.65, 1.30)	0.4195	0.98 (0.87, 1.09)	0.6646
HEI-2015	MV DIAB-adjusted	1	0.91 (0.66, 1.26)	0.89 (0.64, 1.24)	0.77 (0.54, 1.10)	0.93 (0.66, 1.31)	0.441	0.98 (0.87, 1.09)	0.6879
	Invasive ductal carcinoma								
	**Cases/non cases**	463/18127	482/18108	474/18116	478/18112	500/18090			
EDIH	MV BMI-adjusted	1	1.06 (0.93, 1.21)	1.07 (0.94, 1.22)	1.09 (0.95, 1.25)	**1.15 (1.00, 1.33)**	0.0592	**1.04 (0.99, 1.08)**	0.1274
EDIH	MV DIAB-adjusted	1	1.07 (0.94, 1.22)	1.08 (0.94, 1.24)	**1.11 (0.96, 1.27)**	**1.20 (1.04, 1.38)**	0.0137	**1.05 (1.00, 1.10)**	0.0329
	**Cases/non cases**	489/18101	512/18078	465/18125	492/18098	439/18151			
EDIP	MV BMI-adjusted	1	1.07 (0.94, 1.22)	1.00 (0.86, 1.15)	1.10 (0.94, 1.28)	1.02 (0.86, 1.21)	0.7284	**1.04 (0.98, 1.10)**	0.2443
EDIP	MV DIAB-adjusted	1	1.08 (0.94, 1.22)	1.00 (0.87, 1.16)	1.11 (0.95, 1.30)	1.05 (0.89, 1.25)	0.4926	**1.05 (0.99, 1.10)**	0.1248
	**Cases/non cases**	459/18130	485/18105	450/18139	506/18084	497/18092			

HEI-2015	MV BMI-adjusted	1	1.02 (0.90, 1.16)	0.95 (0.83, 1.08)	1.07 (0.94, 1.22)	1.07 (0.93, 1.22)	0.2826	1.03 (0.98, 1.07)	0.2598
HEI-2015	MV DIAB-adjusted	1	1.02 (0.89, 1.16)	0.93 (0.82, 1.06)	1.05 (0.92, 1.19)	1.04 (0.90, 1.18)	0.5455	1.01 (0.97, 1.06)	0.5239
	Invasive lobular carcinoma								
	**Cases/non cases**	87/18630	81/18637	65/18653	68/18650	81/18637			
EDIH	MV BMI-adjusted	1	1.03 (0.76, 1.41)	0.88 (0.62, 1.23)	0.95 (0.67, 1.34)	1.18 (0.84, 1.66)	0.4634	**1.09 (0.98, 1.22)**	0.1108
EDIH	MV DIAB-adjusted	1	**1.04 (0.76, 1.42)**	**0.88 (0.63, 1.24)**	**0.97 (0.69, 1.37)**	**1.22 (0.87, 1.72)**	**0.3406**	**1.10 (0.99, 1.23)**	**0.0697**
	**Cases/non cases**	96/18621	62/18656	89/18629	64/18654	71/18647			
EDIP	MV BMI-adjusted	1	0.69 (0.49, 0.97)	1.04 (0.75, 1.46)	0.80 (0.54, 1.16)	0.96 (0.65, 1.44)	0.988	0.99 (0.86, 1.14)	0.8901
EDIP	MV DIAB-adjusted	1	0.70 (0.49, 0.98)	1.05 (0.76, 1.47)	0.81 (0.55, 1.18)	0.99 (0.67, 1.48)	0.9039	1.00 (0.87, 1.16)	0.9977
	**Cases/non cases**	80/18637	57/18660	77/18641	82/18635	86/18631			
HEI-2015	MV BMI-adjusted	1	0.67 (0.48, 0.94)	0.88 (0.64, 1.21)	0.91 (0.66, 1.25)	0.94 (0.68, 1.30)	0.8107	1.02 (0.92, 1.14)	0.7076
HEI-2015	MV DIAB-adjusted	1	0.66 (0.47, 0.93)	0.87 (0.63, 1.19)	0.89 (0.65, 1.23)	0.91 (0.66, 1.26)	0.9587	1.01 (0.90, 1.13)	0.8594
	Localized								
	**Cases/non cases**	622/17931	633/17921	580/17974	582/17972	615/17938			
EDIH	MV BMI-adjusted	1	1.08 (0.96, 1.21)	1.02 (0.91, 1.16)	1.05 (0.93, 1.18)	**1.14 (1.00, 1.29)**	0.0845	**1.04 (1.00, 1.08)**	0.034
EDIH	MV DIAB-adjusted	1	1.08 (0.97, 1.21)	1.03 (0.92, 1.16)	1.07 (0.94, 1.20)	**1.18 (1.04, 1.33)**	0.0203	**1.06 (1.02, 1.10)**	0.0064
	**Cases/non cases**	630/17923	658/17896	597/17957	606/17948	541/18012			
EDIP	MV BMI-adjusted	1	1.13 (1.00, 1.27)	1.07 (0.94, 1.22)	1.15 (1.01, 1.32)	1.11 (0.96, 1.29)	0.1732	**1.04 (0.99, 1.10)**	0.125
EDIP	MV DIAB-adjusted	1	1.13 (1.01, 1.28)	1.08 (0.95, 1.23)	**1.17 (1.02, 1.34)**	**1.14 (0.98, 1.32)**	0.0859	**1.05 (1.00, 1.11)**	0.0564
	**Cases/non cases**	584/17969	595/17958	597/17956	598/17955	658/17895			
HEI-2015	MV BMI-adjusted	1	0.98 (0.87, 1.09)	0.96 (0.86, 1.08)	0.95 (0.85, 1.07)	1.05 (0.94, 1.19)	0.5398	1.01 (0.97, 1.05)	0.5121
HEI-2015	MV DIAB-adjusted	1	0.97 (0.86, 1.08)	0.95 (0.84, 1.07)	0.94 (0.83, 1.06)	1.03 (0.91, 1.16)	0.8664	1.00 (0.97, 1.04)	0.8458
	Regional/distant								
	**Cases/non cases**	219/18440	230/18430	199/18460	215/18445	229/18430			
EDIH	MV BMI-adjusted	1	1.09 (0.90, 1.31)	0.97 (0.79, 1.19)	1.06 (0.86, 1.30)	1.13 (0.92, 1.39)	0.329	0.98 (0.92, 1.05)	0.6611
EDIH	MV DIAB-adjusted	1	1.09 (0.90, 1.32)	0.98 (0.80, 1.21)	1.09 (0.89, 1.34)	**1.20 (0.97, 1.47)**	0.1095	1.01 (0.94, 1.08)	0.8315
	**Cases/non cases**	234/18425	220/18440	224/18435	219/18441	195/18464			

EDIP	MV BMI-adjusted	1	0.99 (0.81, 1.21)	1.06 (0.85, 1.31)	1.08 (0.86, 1.36)	1.01 (0.79, 1.30)	0.7094	1.01 (0.93, 1.11)	0.7472
EDIP	MV DIAB-adjusted	1	1.00 (0.82, 1.22)	1.07 (0.86, 1.32)	1.11 (0.88, 1.39)	1.06 (0.83, 1.36)	0.4586	1.03 (0.95, 1.13)	0.477
	**Cases/non cases**	226/18433	216/18443	201/18458	220/18439	229/18430			
HEI-2015	MV BMI-adjusted	1	0.94 (0.78, 1.13)	0.87 (0.72, 1.05)	0.95 (0.78, 1.16)	1.01 (0.83, 1.23)	0.9431	1.00 (0.94, 1.06)	0.9592
HEI-2015	MV DIAB-adjusted	1	0.92 (0.77, 1.12)	0.85 (0.70, 1.03)	0.92 (0.76, 1.12)	0.96 (0.79, 1.17)	0.6921	0.98 (0.92, 1.05)	0.5934
	**Endometrial cancer**								
	**Cases/non cases**	74/8946	88/8932	79/8942	73/8947	89/8931			
EDIH	MV BMI-adjusted	1	1.23 (0.89, 1.69)	1.12 (0.80, 1.57)	1.04 (0.73, 1.48)	1.18 (0.84, 1.68)	0.5845	1.02 (0.92, 1.13)	0.7764
EDIH	MV DIAB-adjusted	1	1.30 (0.94, 1.79)	1.24 (0.89, 1.73)	1.23 (0.87, 1.75)	1.60 (1.14, 2.26)	0.0154	**1.12 (1.01, 1.24)**	0.0297
	**Cases/non cases**	81/8939	93/8927	67/8954	84/8936	78/8942			
EDIP	MV BMI-adjusted	1	1.17 (0.85, 1.62)	0.87 (0.60, 1.25)	1.12 (0.77, 1.64)	1.11 (0.74, 1.68)	0.7238	1.06 (0.92, 1.22)	0.4114
EDIP	MV DIAB-adjusted	1	1.22 (0.88, 1.68)	0.94 (0.65, 1.36)	**1.27 (0.87, 1.85)**	**1.38 (0.91, 2.07)**	0.1605	**1.15 (1.00, 1.33)**	0.0524
	**Cases/non cases**	67/8953	84/8936	98/8923	81/8939	73/8947			
HEI-2015	MV BMI-adjusted	1	1.23 (0.89, 1.71)	1.41 (1.03, 1.94)	1.17 (0.84, 1.63)	1.09 (0.77, 1.55)	0.7006	1.02 (0.92, 1.14)	0.6501
HEI-2015	MV DIAB-adjusted	1	1.15 (0.84, 1.59)	1.26 (0.92, 1.73)	1.00 (0.72, 1.40)	0.87 (0.62, 1.23)	0.2891	0.95 (0.85, 1.05)	0.3008
	Endometrioid								
	**Cases/non cases**	49/9148	50/9147	53/9145	52/9145	62/9135			
EDIH	MV BMI-adjusted	1	1.08 (0.72, 1.61)	1.16 (0.77, 1.76)	1.15 (0.75, 1.75)	1.25 (0.82, 1.91)	0.2965	1.05 (0.93, 1.19)	0.449
EDIH	MV DIAB-adjusted	1	1.14 (0.76, 1.72)	1.30 (0.86, 1.95)	1.36 (0.90, 2.08)	**1.72 (1.13, 2.60)**	0.0076	**1.16 (1.03, 1.32)**	0.0157
	**Cases/non cases**	54/9143	59/9138	42/9156	55/9142	56/9141			
EDIP	MV BMI-adjusted	1	1.13 (0.76, 1.69)	0.83 (0.52, 1.30)	1.11 (0.70, 1.76)	1.20 (0.73, 1.96)	0.5588	1.11 (0.94, 1.32)	0.225
EDIP	MV DIAB-adjusted	1	1.19 (0.80, 1.78)	0.90 (0.57, 1.43)	1.27 (0.80, 2.02)	1.51 (0.92, 2.45)	0.1264	**1.22 (1.02, 1.45)**	0.0264
	**Cases/non cases**	48/9149	53/9144	66/9132	54/9143	45/9152			
HEI-2015	MV BMI-adjusted	1	1.10 (0.74, 1.62)	1.35 (0.92, 1.98)	1.10 (0.74, 1.64)	0.97 (0.63, 1.48)	0.9668	1.00 (0.88, 1.14)	0.9369
HEI-2015	MV DIAB-adjusted	1	1.02 (0.69, 1.52)	1.20 (0.82, 1.76)	0.94 (0.63, 1.40)	0.76 (0.50, 1.17)	0.2025	0.93 (0.82, 1.05)	0.2294
	Non-endometrioid								
	**Cases/non cases**	25/9181	39/9167	25/9181	21/9185	27/9179			

EDIH	MV BMI-adjusted	1	1.50 (0.90, 2.53)	0.98 (0.54, 1.75)	0.84 (0.45, 1.56)	1.03 (0.56, 1.90)	0.5258	0.93 (0.78, 1.12)	0.4568
EDIH	MV DIAB-adjusted	1	1.58 (0.94, 2.65)	1.07 (0.60, 1.92)	0.96 (0.52, 1.80)	1.35 (0.74, 2.45)	0.7732	1.02 (0.85, 1.22)	0.8641
	**Cases/non cases**	27/9179	33/9173	26/9180	29/9177	22/9184			
EDIP	MV BMI-adjusted	1	1.17 (0.68, 2.04)	0.95 (0.51, 1.76)	1.07 (0.56, 2.05)	0.85 (0.41, 1.75)	0.629	0.94 (0.74, 1.20)	0.6326
EDIP	MV DIAB-adjusted	1	1.22 (0.70, 2.11)	1.02 (0.55, 1.90)	1.20 (0.62, 2.29)	1.03 (0.50, 2.12)	0.9473	1.01 (0.79, 1.29)	0.9298
	**Cases/non cases**	19/9187	31/9175	31/9175	27/9179	29/9177			
HEI-2015	MV BMI-adjusted	1	1.59 (0.90, 2.83)	1.55 (0.87, 2.77)	1.37 (0.75, 2.50)	1.51 (0.83, 2.77)	0.3243	1.09 (0.91, 1.30)	0.3529
HEI-2015	MV DIAB-adjusted	1	1.51 (0.85, 2.68)	1.41 (0.79, 2.52)	1.19 (0.65, 2.17)	1.22 (0.67, 2.24)	0.8454	1.01 (0.85, 1.21)	0.9071
	**Ovarian cancer**								
	**Cases/non cases**	60/8963	52/8972	54/8970	41/8983	53/8970			
EDIH	MV BMI-adjusted	1	0.94 (0.64, 1.39)	1.03 (0.70, 1.53)	0.85 (0.55, 1.30)	1.16 (0.76, 1.75)	0.6223	1.02 (0.90, 1.16)	0.7549
EDIH	MV DIAB-adjusted	1	0.94 (0.64, 1.37)	1.01 (0.68, 1.49)	0.82 (0.53, 1.25)	1.10 (0.73, 1.66)	0.7927	1.00 (0.88, 1.14)	0.9391
	**Cases/non cases**	64/8959	63/8961	44/8980	46/8978	43/8980			
EDIP	MV BMI-adjusted	1	1.02 (0.70, 1.49)	0.76 (0.49, 1.18)	0.85 (0.53, 1.35)	0.88 (0.53, 1.46)	0.4352	1.00 (0.83, 1.19)	0.9563
EDIP	MV DIAB-adjusted	1	1.02 (0.70, 1.50)	0.75 (0.48, 1.16)	0.84 (0.53, 1.34)	0.87 (0.52, 1.43)	0.3945	0.98 (0.83, 1.17)	0.8592
	**Cases/non cases**	47/8976	45/8979	42/8982	69/8955	57/8966			
HEI-2015	MV BMI-adjusted	1	0.93 (0.62, 1.40)	0.85 (0.56, 1.30)	1.38 (0.94, 2.02)	1.10 (0.73, 1.66)	0.2257	1.07 (0.94, 1.22)	0.2929
HEI-2015	MV DIAB-adjusted	1	0.93 (0.62, 1.40)	0.85 (0.56, 1.30)	1.38 (0.94, 2.02)	1.10 (0.74, 1.66)	0.2209	1.07 (0.94, 1.22)	0.2874
	Serous								
	**Cases/non cases**	27/9177	25/9180	23/9182	15/9190	24/9180			
EDIH	MV BMI-adjusted	1	1.06 (0.60, 1.86)	1.07 (0.59, 1.93)	0.76 (0.38, 1.49)	1.29 (0.69, 2.40)	0.6587	1.10 (0.90, 1.33)	0.344
EDIH	MV DIAB-adjusted	1	1.04 (0.60, 1.83)	1.04 (0.58, 1.88)	0.74 (0.38, 1.44)	1.23 (0.67, 2.27)	0.7553	1.08 (0.89, 1.31)	0.428
	**Cases/non cases**	28/9176	35/9170	20/9185	10/9195	21/9183			
EDIP	MV BMI-adjusted	1	1.38 (0.80, 2.40)	0.89 (0.46, 1.72)	0.49 (0.21, 1.12)	1.15 (0.54, 2.45)	0.5784	0.98 (0.75, 1.27)	0.8558
EDIP	MV DIAB-adjusted	1	1.38 (0.80, 2.39)	0.87 (0.45, 1.69)	0.48 (0.21, 1.11)	1.14 (0.54, 2.41)	0.551	0.96 (0.74, 1.25)	0.7837
	**Cases/non cases**	23/9181	19/9186	21/9184	28/9177	23/9181			

HEI-2015	MV BMI-adjusted	1	0.80 (0.44, 1.48)	0.86 (0.47, 1.56)	1.12 (0.64, 2.00)	0.89 (0.48, 1.65)	0.9317	0.97 (0.80, 1.18)	0.7414
HEI-2015	MV DIAB-adjusted	1	0.81 (0.44, 1.49)	0.88 (0.48, 1.60)	1.14 (0.65, 2.02)	0.92 (0.50, 1.69)	0.8504	0.98 (0.81, 1.19)	0.8239
	Non-Serous								
	**Cases/non cases**	33/9171	27/9177	31/9173	26/9178	29/9175			
EDIH	MV BMI-adjusted	1	0.85 (0.51, 1.44)	1.01 (0.60, 1.69)	0.91 (0.52, 1.58)	1.07 (0.61, 1.88)	0.7619	0.96 (0.80, 1.15)	0.6449
EDIH	MV DIAB-adjusted	1	0.85 (0.50, 1.43)	0.98 (0.58, 1.64)	0.87 (0.50, 1.51)	1.01 (0.58, 1.76)	0.9384	0.94 (0.79, 1.12)	0.5038
	**Cases/non cases**	36/9168	28/9176	24/9180	36/9168	22/9182			
EDIP	MV BMI-adjusted	1	0.75 (0.44, 1.28)	0.66 (0.36, 1.18)	1.03 (0.58, 1.85)	0.69 (0.35, 1.37)	0.5354	1.01 (0.80, 1.28)	0.924
EDIP	MV DIAB-adjusted	1	0.76 (0.45, 1.29)	0.65 (0.36, 1.17)	1.02 (0.57, 1.83)	0.68 (0.34, 1.33)	0.4836	1.00 (0.79, 1.26)	0.9999
	**Cases/non cases**	23/9181	27/9177	21/9183	41/9163	34/9170			
HEI-2015	MV BMI-adjusted	1	1.15 (0.66, 2.00)	0.88 (0.48, 1.60)	1.68 (0.99, 2.86)	1.36 (0.78, 2.37)	0.1158	**1.17 (0.98, 1.39)**	0.086
HEI-2015	MV DIAB-adjusted	1	1.14 (0.65, 1.99)	0.87 (0.48, 1.58)	1.66 (0.98, 2.82)	1.32 (0.76, 2.31)	0.1333	**1.16 (0.97, 1.38)**	0.0993
	**Lung cancer**								
	**Cases/non cases**	482/20722	408/20797	410/20795	417/20788	399/20805			
EDIH	MV BMI-adjusted	1	0.94 (0.82, 1.08)	0.98 (0.85, 1.12)	1.03 (0.89, 1.18)	0.92 (0.79, 1.06)	0.4724	0.96 (0.92, 1.01)	0.1257
EDIH	MV DIAB-adjusted	1	0.96 (0.84, 1.10)	0.99 (0.86, 1.14)	1.03 (0.89, 1.19)	**0.86 (0.74, 1.00)**	0.1208	0.94 (0.90, 0.99)	0.0128
	**Cases/non cases**	547/20657	464/20741	397/20808	377/20828	331/20873			
EDIP	MV BMI-adjusted	1	1.05 (0.92, 1.21)	0.98 (0.85, 1.14)	1.05 (0.89, 1.23)	1.04 (0.87, 1.25)	0.6951	0.98 (0.92, 1.04)	0.4603
EDIP	MV DIAB-adjusted	1	1.06 (0.92, 1.21)	0.99 (0.85, 1.15)	1.05 (0.89, 1.23)	0.96 (0.80, 1.14)	0.6906	0.96 (0.90, 1.02)	0.1411
	**Cases/non cases**	520/20684	439/20765	415/20789	387/20817	354/20850			
HEI-2015	MV BMI-adjusted	1	0.97 (0.85, 1.10)	0.99 (0.86, 1.13)	0.96 (0.83, 1.10)	0.89 (0.77, 1.03)	0.1678	**0.95 (0.91, 1.00)**	0.0293
HEI-2015	MV DIAB-adjusted	1	1.02 (0.89, 1.15)	1.05 (0.92, 1.20)	1.04 (0.91, 1.20)	1.01 (0.87, 1.17)	0.7406	0.99 (0.95, 1.04)	0.6553
	Small cell								
	**Cases/non cases**	25/21248	32/21242	26/21248	29/21245	26/21247			
EDIH	MV BMI-adjusted	1	1.42 (0.82, 2.43)	1.02 (0.57, 1.82)	1.13 (0.63, 2.01)	0.78 (0.42, 1.43)	0.2686	0.98 (0.83, 1.17)	0.8577
EDIH	MV DIAB-adjusted	1	1.46 (0.85, 2.50)	1.08 (0.60, 1.91)	1.19 (0.67, 2.12)	0.78 (0.42, 1.42)	0.2568	0.97 (0.83, 1.15)	0.7486
	**Cases/non cases**	31/21242	38/21236	23/21251	28/21246	18/21255			

EDIP	MV BMI-adjusted	1	1.54 (0.92, 2.60)	0.92 (0.49, 1.70)	1.26 (0.67, 2.38)	0.81 (0.38, 1.69)	0.5673	0.99 (0.79, 1.25)	0.9444
EDIP	MV DIAB-adjusted	1	1.55 (0.92, 2.61)	0.98 (0.53, 1.81)	1.32 (0.70, 2.48)	0.78 (0.37, 1.63)	0.5391	0.98 (0.78, 1.23)	0.8707
	**Cases/non cases**	33/21240	27/21246	32/21241	23/21250	23/21250			
HEI-2015	MV BMI-adjusted	1	1.22 (0.72, 2.04)	1.81 (1.09, 3.01)	1.52 (0.87, 2.69)	**1.82 (1.01, 3.28)**	0.0241	**1.25 (1.04, 1.50)**	0.0162
HEI-2015	MV DIAB-adjusted	1	1.25 (0.75, 2.09)	1.93 (1.16, 3.19)	1.66 (0.94, 2.91)	**2.06 (1.15, 3.69)**	0.0065	**1.29 (1.08, 1.54)**	0.0053
	Non small cell								
	**Cases/non cases**	249/20988	183/21055	204/21033	225/21013	198/21039			
EDIH	MV BMI-adjusted	1	0.82 (0.67, 1.00)	0.95 (0.78, 1.16)	1.09 (0.89, 1.32)	0.93 (0.75, 1.14)	0.8739	0.97 (0.91, 1.03)	0.3285
EDIH	MV DIAB-adjusted	1	0.83 (0.68, 1.01)	0.96 (0.79, 1.17)	1.10 (0.90, 1.33)	0.88 (0.71, 1.08)	0.7139	0.95 (0.89, 1.01)	0.1220
	**Cases/non cases**	275/20962	218/21020	205/21032	192/21046	169/21068			
EDIP	MV BMI-adjusted	1	0.97 (0.81, 1.19)	1.01 (0.82, 1.25)	1.07 (0.85, 1.34)	1.08 (0.84, 1.39)	0.4556	1.00 (0.92, 1.09)	0.9498
EDIP	MV DIAB-adjusted	1	0.98 (0.81, 1.19)	1.01 (0.82, 1.25)	1.06 (0.84, 1.33)	0.98 (0.77, 1.26)	0.8908	0.97 (0.89, 1.06)	0.5158
	**Cases/non cases**	251/20985	219/21018	207/21030	197/21040	184/21053			
HEI-2015	MV BMI-adjusted	1	0.97 (0.81, 1.17)	0.96 (0.80, 1.17)	0.94 (0.77, 1.14)	0.88 (0.72, 1.08)	0.2366	0.94 (0.89, 1.01)	0.0895
HEI-2015	MV DIAB-adjusted	1	1.01 (0.84, 1.21)	1.02 (0.84, 1.23)	1.01 (0.83, 1.23)	0.98 (0.80, 1.20)	0.9126	0.98 (0.92, 1.05)	0.5628

a.Values presented are hazard ratios (HR) and 95% confidence intervals (95% CI). HRs were derived from multivariable BMI-adjusted or multivariable DIAB-adjusted Cox proportional hazards regression models adjusted for body mass indes and baseline diabetes, respectively, then further adjusted for the following baseline covariates:age at enrollment, physical activity, race and ethnicity, educational level, family history of cancer, number of hormones used, comorbidity score, baseline cardiovascular disease status, baseline lung disease (we excluded baseline lung disease cases in lung cancer analyses), number of supplements used, non-steroidal anti-inflammatory drug use, hormone therapy study arm (Not randomized to HRT, E-alone intervention, E-alone control, E+P intervention, E+P control), baseline hormone therapy ever, oral contraceptive duration, pack-years of smoking, coffee/tea, and total alcohol intake. Colorectal cancer and subtype analyses were additionally adjusted for colorectal cancer screening. Invasive breast cancer and subtype analyses were additionally adjusted for hysterectomy age, months of breast-feeding, age at menopause, mammogram ever, parity, bilateral oophorectomy, passive smoking, Gail 5-year risk score. Endometrial cancer and ovarian cancer analyses were additionally adjusted for age at first birth, age at menarche, age at menopause, months of breast-feeding, and parity. Ovarian cancer analyses were further adjusted for tubal ligation. Lung cancer analyses were additionally adjusted for smoking status (current, former, never smokers) and passive smoking.

b.Cancer sites were defined as follows: total cancer was the first occurrence of any cancer except non-melanoma skin cancer. Proximal colon - cecum, ascending colon, hepatic flexure of colon, transverse colon (ICD site codes 18.0,18.2,18.3, 18.4); distal colon - splenic flexure of colon, descending colon, sigmoid colon (ICD site codes 18.5, 18.6,18.7); Luminal A breast cancer - ER+ and/or PR+ , HER2-; luminal B breast cancer - ER+ and/or PR+ , HER2+; type 1 endometrial cancer - Adenocarcinoma NOS, endometrioid carcinoma (SEER ICD histology codes 8140/3,8380/3); type II endometrial cancer - cases other than type I endometrial cancer cases; serous ovarian cancer - Undifferentiated carcinoma, NOS, anaplastic carcinoma, NOS, pleomorphic carcinoma, papillary carcinoma NOS, transitional cell carcinoma NOS, papillary transitional cell carcinoma, non-invasive, papillary transitional cell carcinoma, papillary adencarcinoma, NOS, serous cystadenocarcinoma/borderline, serous cystadenocarcinoma, NOS, serous cystadenoma, borderline malignancy, serous cystadenoma borderline malignancy, papillary cystadenocarcinoma NOS, papillary cystadenocarcinoma serous, serous surface papillary carcinoma, serous papillary cystic tumor of borderline malignancy, papillary cystadenoma serous, borderline malignancy,serous adenocarcinofibroma (SEER ICD histology codes 8020/3, 8021/3, 8022/3, 8050/3, 8120/3, 8130/2, 8130/3, 8260/3, 8441/1, 8441/3, 8442/1, 8442/3, 8450/3, 8460/3, 8461/3, 8462/1, 9014/3); non-serous ovarian cancer - Endometrioid carcinoma, endometrioid adenofibroma, malignant, endometrioid adenocarcinoma, secretory variant, endometrioid adenocarcinoma, ciliated cell variant, clear cell adenocarcinoma, NOS, Mucinous cystadenocarcinoma, NOS, mucinous cystadenocarcinoma, papillary, mucinous cystadenoma borderline malignancy, mucous adenocarcinoma, mullerian mixed tumor, mixed cell adenocarcinoma, brenner tumour borderline malignancy, brenner tumor malignant (SEER ICD histology code 8380/3, 8381/3, 8382/3, 383/3,8310/3,8470/3,8471/3,8472/1,8480/3,8950/3,8323/3,9000/1,9000/3; small-cell lung cancer - Small cell carcinoma NOS, oat cell carcinoma, small cell carcinoma fusiform cell, small cell carcinoma intermediate cell, small cell-large cell carcinoma (SEER ICD histology code 8041/3, 8042/3,8043/3,8044/3, 8045/3); non-small cell lung cancer - Non-small cell carcinoma, squamous cell carcinoma nos,

adenocarcinoma NOS, bronchiolo-alveolar adenocarcinoma, large cell carcinoma NOS, adenosquamous carcinoma, carcinoid tumour, NOS (except appendix m-8240/1), mesothelioma malignant (SEER ICD histology code 8046/3, 8070/3, 8140/3, 8250/3, 8012/3, 8560/3, 8240/3, 9050/3).

c.The p value for linear trend was estimated in the same multivariable-adjusted models by assigning the quintile-specific median value of each dietary pattern to all participants in the quintile and modelling as an ordinal variable.

d.Dietary score was input in the corresponding multivariable BMI adjusted or multivariable DIAB adjusted models as a continuous variable.

Supplementary Table S6. Multivariable-adjusted associations of dietary patterns with specific cancers in body mass index (kg/ m^2) subgroups[a,b]

Dietary pattern[c]/ cancer site	**1-SD increment in dietary score**	**P-value**	**1-SD increment in dietary score**	**P-value**	**1-SD increment in dietary score**	**P-value**	**P interaction[d]**
	Normal weight (18.5-24.9)		*Overweight (25-29.9)*		*Obese (>30)*		
**Total cancer (except non-melanoma skin cancer)**							
EDIH	**1.04 (1.01, 1.06)**	0.0141	1.00 (0.98, 1.03)	0.7771	1.01 (0.98, 1.04)	0.4243	0.5376
EDIP	**1.03 (0.99, 1.07)**	0.0993	**1.03 (0.99, 1.07)**	0.1032	**1.03 (0.99, 1.07)**	0.1019	**0.0033**
HEI2015	**0.97 (0.95, 1.00)**	0.0234	**0.97 (0.95, 1.00)**	0.0433	0.98 (0.96, 1.01)	0.2341	0.3741
**Colorectal cancer**							
EDIH	1.02 (0.92, 1.14)	0.6567	**1.09 (0.98, 1.20)**	0.1015	**1.07 (0.98, 1.17)**	0.1507	0.7988
EDIP	1.07 (0.94, 1.22)	0.3342	**1.10 (0.98, 1.27)**	0.1126	1.04 (0.91, 1.18)	0.5656	0.9436
HEI2015	**0.89 (0.81, 0.98)**	0.0127	0.93 (0.85, 1.03)	0.1538	1.00 (0.91, 1.10)	0.9321	**0.0296**
**Colon Cancer**							
EDIH	1.02 (0.91, 1.14)	0.7788	**1.12 (1.00, 1.24)**	0.0499	**1.09 (0.98, 1.20)**	0.1049	0.7943
EDIP	1.07 (0.93, 1.23)	0.3236	**1.14 (0.99, 1.32)**	0.0921	1.06 (0.92, 1.22)	0.4031	0.9748
HEI2015	0.91 (0.82, 1.00)	0.0581	0.92 (0.83, 1.02)	0.0992	0.98 (0.89, 1.09)	0.7496	0.1153
Proximal colon cancer							
EDIH	1.10 (0.95, 1.27)	0.2195	**1.14 (0.99, 1.31)**	0.0728	**1.10 (0.97, 1.25)**	0.1262	0.7014
EDIP	1.06 (0.88, 1.28)	0.5074	1.11 (0.92, 1.33)	0.2892	1.08 (0.90, 1.29)	0.4238	0.8933
HEI2015	0.90 (0.79, 1.02)	0.102	0.96 (0.84, 1.10)	0.6072	0.96 (0.84, 1.09)	0.5072	0.4318
Distal colon and rectal cancer							
EDIH	1.06 (0.89, 1.26)	0.5374	0.99 (0.84, 1.17)	0.9022	1.05 (0.90, 1.22)	0.5485	0.7693
EDIP	1.09 (0.87, 1.36)	0.4542	1.06 (0.86, 1.32)	0.5753	1.10 (0.90, 1.36)	0.3438	0.5558
HEI2015	0.97 (0.83, 1.14)	0.7116	0.90 (0.78, 1.04)	0.1646	0.98 (0.83, 1.14)	0.7579	0.3594
Intestinal polyps							
EDIH	**1.09 (1.05, 1.13)**	<.0001	1.03 (0.99, 1.07)	0.1225	**1.06 (1.02, 1.10)**	0.0012	0.2815
EDIP	1.04 (0.99, 1.10)	0.1383	**1.04 (0.99, 1.10)**	0.0832	**1.05 (1.00, 1.10)**	0.0574	0.7083
HEI2015	**0.93 (0.89, 0.96)**	<.0001	**0.95 (0.91, 0.98)**	**0.0025**	**0.96 (0.93, 1.00)**	0.0647	**0.0238**
**Invasive breast cancer**							
EDIH	**1.10 (1.03, 1.16)**	0.0025	1.00 (0.95, 1.06)	0.9491	1.01 (0.95, 1.06)	0.7766	0.8284
EDIP	**1.05 (0.98, 1.14)**	0.1767	1.00 (0.93, 1.08)	0.9395	1.06 (0.98, 1.14)	0.1426	0.0617
HEI2015	1.03 (0.98, 1.09)	0.2467	0.98 (0.92, 1.03)	0.3785	1.00 (0.94, 1.05)	0.8552	0.4120
ER+							

EDIH	**1.08 (1.01, 1.16)**	0.0181	0.98 (0.92, 1.05)	0.5898	1.00 (0.94, 1.06)	0.8793	0.6604
EDIP	1.05 (0.96, 1.14)	0.2976	1.00 (0.92, 1.09)	0.9347	1.00 (0.92, 1.09)	0.9481	0.5558
HEI2015	1.04 (0.98, 1.11)	0.2145	0.99 (0.93, 1.06)	0.8108	1.02 (0.96, 1.09)	0.5236	0.6819
ER-							
EDIH	**1.23 (1.06, 1.44)**	0.0076	1.06 (0.90, 1.24)	0.5151	1.06 (0.90, 1.26)	0.4533	0.6864
EDIP	1.16 (0.95, 1.43)	0.1427	0.94 (0.76, 1.17)	0.5998	**1.26 (1.01, 1.58)**	0.0385	**0.0101**
HEI2015	1.03 (0.89, 1.19)	0.704	0.92 (0.79, 1.07)	0.2842	0.91 (0.77, 1.09)	0.3104	0.1146
PR+							
EDIH	**1.09 (1.02, 1.18)**	0.0126	1.00 (0.93, 1.07)	0.9161	0.99 (0.93, 1.06)	0.8215	0.3277
EDIP	1.05 (0.96, 1.15)	0.2868	1.02 (0.93, 1.12)	0.6592	0.99 (0.90, 1.08)	0.7517	0.8963
HEI2015	1.02 (0.95, 1.09)	0.5582	0.99 (0.93, 1.06)	0.8494	1.03 (0.96, 1.10)	0.3961	0.7424
PR-							
EDIH	**1.13 (1.00, 1.26)**	0.0404	0.99 (0.88, 1.11)	0.8224	1.05 (0.93, 1.18)	0.4551	0.1652
EDIP	1.11 (0.95, 1.28)	0.1796	0.92 (0.79, 1.07)	0.2673	**1.17 (1.00, 1.38)**	0.0566	**0.0052**
HEI2015	1.07 (0.96, 1.19)	0.2306	0.96 (0.85, 1.07)	0.4301	0.93 (0.82, 1.05)	0.2337	**0.0280**
HER2+							
EDIH	1.10 (0.92, 1.32)	0.3002	0.98 (0.82, 1.18)	0.8322	1.13 (0.96, 1.34)	0.1458	0.2537
EDIP	1.16 (0.91, 1.46)	0.2316	0.96 (0.76, 1.22)	0.7675	1.10 (0.86, 1.39)	0.4591	0.0995
HEI2015	1.02 (0.86, 1.21)	0.8531	1.03 (0.86, 1.22)	0.7729	0.91 (0.76, 1.10)	0.3384	0.5922
HER2-							
EDIH	**1.11 (1.04, 1.19)**	**0.0024**	0.99 (0.93, 1.06)	0.8484	0.99 (0.92, 1.05)	0.6722	0.5530
EDIP	1.05 (0.96, 1.14)	0.2926	0.99 (0.91, 1.08)	0.7593	1.05 (0.96, 1.15)	0.2494	0.1834
HEI2015	1.03 (0.97, 1.10)	0.3145	0.98 (0.92, 1.05)	0.6213	1.00 (0.94, 1.07)	0.8942	0.3331
Luminal A							
EDIH	**1.08 (1.01, 1.17)**	**0.0263**	0.99 (0.92, 1.06)	0.6882	0.97 (0.91, 1.04)	0.4137	0.5215
EDIP	1.03 (0.94, 1.13)	0.517	1.00 (0.91, 1.09)	0.9643	1.03 (0.94, 1.12)	0.5779	0.4386
HEI2015	1.02 (0.96, 1.10)	0.4717	0.98 (0.92, 1.05)	0.6428	1.02 (0.95, 1.09)	0.6283	0.5947
Luminal B							
EDIH	1.10 (0.89, 1.36)	0.3834	0.99 (0.80, 1.24)	0.9444	**1.20 (0.99, 1.46)**	0.0586	0.1474
EDIP	1.16 (0.88, 1.53)	0.2815	0.96 (0.72, 1.27)	0.7526	1.07 (0.80, 1.42)	0.6469	0.1114
HEI2015	1.06 (0.86, 1.31)	0.5762	1.08 (0.88, 1.34)	0.4687	0.94 (0.76, 1.16)	0.5591	0.7103
Triple negative							
EDIH	**1.30 (1.08, 1.57)**	0.0061	1.06 (0.86, 1.30)	0.6015	1.15 (0.94, 1.41)	0.1815	0.7083
EDIP	1.18 (0.91, 1.52)	0.2086	0.87 (0.67, 1.13)	0.2986	**1.35 (1.02, 1.79)**	0.0346	**0.0305**
HEI2015	1.06 (0.88, 1.28)	0.511	0.98 (0.81, 1.19)	0.8682	0.86 (0.69, 1.08)	0.1969	0.1262
Invasive ductal carcinoma							

EDIH	**1.08 (0.99, 1.17)**	0.0727	1.03 (0.96, 1.12)	0.4126	1.02 (0.95, 1.10)	0.5894	0.7604
EDIP	1.00 (0.90, 1.12)	0.9379	1.03 (0.93, 1.14)	0.5948	**1.08 (0.97, 1.19)**	0.1577	**0.0514**
HEI2015	**1.07 (0.99, 1.15)**	0.0932	0.96 (0.89, 1.04)	0.294	**1.05 (0.97, 1.14)**	0.2301	0.8516
Invasive lobular carcinoma							
EDIH	**1.32 (1.09, 1.58)**	0.004	1.15 (0.96, 1.37)	0.1194	0.86 (0.70, 1.06)	0.1726	0.2609
EDIP	**1.28 (1.00, 1.64)**	0.0484	0.88 (0.69, 1.11)	0.2806	0.86 (0.65, 1.12)	0.2594	0.7579
HEI2015	1.00 (0.83, 1.21)	0.9953	1.05 (0.87, 1.26)	0.5986	1.05 (0.85, 1.30)	0.6237	1.0000
Localized							
EDIH	**1.12 (1.04, 1.20)**	0.0017	1.01 (0.94, 1.08)	0.772	1.02 (0.96, 1.09)	0.5312	0.8848
EDIP	**1.09 (1.00, 1.20)**	0.0524	1.02 (0.94, 1.12)	0.6195	1.01 (0.92, 1.11)	0.8287	0.4535
HEI2015	1.04 (0.98, 1.12)	0.1957	0.99 (0.92, 1.06)	0.7331	1.01 (0.94, 1.08)	0.7502	0.3566
Regional/distant							
EDIH	0.99 (0.87, 1.12)	0.8224	1.02 (0.91, 1.15)	0.7087	0.96 (0.86, 1.07)	0.4631	0.8065
EDIP	0.93 (0.80, 1.09)	0.3714	0.99 (0.85, 1.15)	0.8577	1.11 (0.96, 1.28)	0.1651	0.1034
HEI2015	1.04 (0.93, 1.17)	0.4515	0.96 (0.86, 1.08)	0.5157	0.97 (0.87, 1.09)	0.6451	0.7506
**Endometrial cancer**			*Overweight/Obese (≥25)*[e]				
EDIH	**1.34 (1.04, 1.72)**	0.0244	1.02 (0.91, 1.15)	0.6696	NA[f]	NA	**0.0020**
EDIP	1.23 (0.87, 1.74)	0.2358	1.08 (0.92, 1.26)	0.3498	NA	NA	**0.0956**
HEI2015	0.94 (0.74, 1.20)	0.6108	1.01 (0.90, 1.13)	0.8869	NA	NA	**0.0338**
Endometrioid			*Overweight/Obese (≥25)*				
EDIH	1.20 (0.86, 1.66)	0.2814	1.09 (0.95, 1.25)	0.211	NA	NA	**0.0395**
EDIP	1.04 (0.68, 1.60)	0.8614	**1.18 (0.98, 1.42)**	0.0854	NA	NA	0.4953
HEI2015	1.18 (0.86, 1.61)	0.3112	0.94 (0.82, 1.08)	0.386	NA	NA	0.5038
**Ovarian cancer**							
EDIH	0.99 (0.77, 1.27)	0.9304	1.10 (0.89, 1.39)	0.3632	0.98 (0.79, 1.22)	0.8754	0.4190
EDIP	0.98 (0.71, 1.35)	0.901	1.26 (0.92, 1.71)	0.1442	0.83 (0.62, 1.12)	0.223	0.3852
HEI2015	0.98 (0.78, 1.23)	0.859	1.08 (0.87, 1.35)	0.4856	1.21 (0.96, 1.53)	0.1134	**0.0637**
**Lung cancer**							
EDIH	0.96 (0.89, 1.03)	0.2553	0.98 (0.91, 1.06)	0.6904	0.96 (0.88, 1.06)	0.4127	0.3580
EDIP	0.97 (0.88, 1.07)	0.5618	0.98 (0.89, 1.09)	0.7086	0.99 (0.87, 1.12)	0.8926	0.3055
HEI2015	0.92 (0.86, 0.99)	0.0317	0.95 (0.87, 1.02)	0.1699	0.99 (0.90, 1.09)	0.9009	0.1649
Small cell							
EDIH	1.02 (0.76, 1.37)	0.8838	0.78 (0.58, 1.06)	0.1082	1.07 (0.80, 1.42)	0.6499	0.4219
EDIP	0.98 (0.64, 1.50)	0.9426	0.79 (0.54, 1.16)	0.2368	1.18 (0.79, 1.76)	0.4215	0.1424
HEI2015	1.11 (0.80, 1.54)	0.5449	1.39 (1.02, 1.90)	0.0368	1.26 (0.92, 1.74)	0.1543	**0.0634**

a. HRs were derived from multivariable-adjusted Cox proportional hazards regression models adjusted for the following baseline covariates: age at enrollment, physical activity, race and ethnicity, educational level, family history of cancer, number of hormones used, comorbidity score, baseline cardiovascular disease status, baseline lung disease, number of supplements used, non-steroidal anti-inflammatory drug use, hormone therapy study arm (Not randomized to HRT, E-alone intervention, E-alone control, E+P intervention, E+P control), baseline hormone therapy ever, oral contraceptive duration, pack-years of smoking, coffee/tea, and total alcohol intake. Colorectal cancer and subtype analyses were additionally adjusted for colorectal cancer screening. Invasive breast cancer and subtype analyses were additionally adjusted for hysterectomy age, months of breast-feeding, age at menopause, mammogram ever, parity, bilateral oophorectomy, passive smoking, Gail 5-year risk score. Endometrial cancer and ovarian cancer analyses were additionally adjusted for age at first birth, age at menarche, age at menopause, months of breast-feeding, and parity. Ovarian cancer analyses were further adjusted for tubal ligation. Lung cancer analyses were additionally adjusted for smoking status (current, former, never smokers) and passive smoking. Dietary score was input in each model as a continuous variable.

b. We presented here the cancer sites that were found to be associated with dietary patterns in the main analysis. Cancer sites were defined as follows: total cancer was the first occurrence of any cancer except non-melanoma skin cancer. Proximal colon - cecum, ascending colon, hepatic flexure of colon, transverse colon (ICD site codes 18.0,18.2,18.3, 18.4); distal colon - splenic flexure of colon, descending colon, sigmoid colon (ICD site codes 18.5, 18.6,18.7); Luminal A breast cancer - ER+ and/or PR+ , HER2-; luminal B breast cancer - ER+ and/or PR+ , HER2+; type 1 endometrial cancer - Adenocarcinoma NOS, endometrioid carcinoma (SEER ICD histology codes 8140/3,8380/3); type II endometrial cancer - cases other than type I endometrial cancer cases; serous ovarian cancer - Undifferentiated carcinoma, NOS, anaplastic carcinoma, NOS, pleomorphic carcinoma, papillary carcinoma NOS, transitional cell carcinoma NOS, papillary transitional cell carcinoma, non-invasive, papillary transitional cell carcinoma, papillary adencarcinoma, NOS, serous cystadenocarcinoma/borderline, serous cystadenocarcinoma, NOS, serous cystadenoma, borderline malignancy, serous cystadenoma borderline malignancy, papillary cystadenocarcinoma NOS, papillary cystadenocarcinoma serous, serous surface papillary carcinoma, serous papillary cystic tumor of borderline malignancy, papillary cystadenoma serous, borderline malignancy,serous adenocarcinofibroma (SEER ICD histology codes 8020/3, 8021/3, 8022/3, 8050/3, 8120/3, 8130/2, 8130/3, 8260/3, 8441/1, 8441/3, 8442/1, 8442/3, 8450/3, 8460/3, 8461/3, 8462/1, 9014/3); non-serous ovarian cancer - Endometrioid carcinoma, endometrioid adenofibroma, malignant, endometrioid adenocarcinoma, secretory variant, endometrioid adenocarcinoma, ciliated cell variant, clear cell adenocarcinoma, NOS, Mucinous cystadenocarcinoma, NOS, mucinous cystadenocarcinoma, papillary, mucinous cystadenoma borderline malignancy, mucous adenocarcinoma, mullerian mixed tumor, mixed cell adenocarcinoma, brenner tumour borderline malignancy, brenner tumor malignant (SEER ICD histology code 8380/3, 8381/3, 8382/3, 383/3,8310/3,8470/3,8471/3,8472/1,8480/3,8950/3,8323/3,9000/1,9000/3; small-cell lung cancer - Small cell carcinoma NOS, oat cell carcinoma, small cell carcinoma fusiform cell, small cell carcinoma intermediate cell, small cell-large cell carcinoma (SEER ICD histology code 8041/3, 8042/3,8043/3,8044/3, 8045/3); non-small cell lung cancer - Non-small cell carcinoma, squamous cell carcinoma nos, adenocarcinoma NOS, bronchiolo-alveolar adenocarcinoma, large cell carcinoma NOS, adenosquamous carcinoma, carcinoid tumour, NOS (except appendix m-8240/1), mesothelioma malignant (SEER ICD histology code 8046/3, 8070/3, 8140/3, 8250/3, 8012/3, 8560/3, 8240/3, 9050/3).

c. EDIH, empirical dietary index for hyperinsulinemia score assessing the ability of the dietary pattern to contribute to insulin hypersecretion - higher EDIH scores reflect more hyperinsulinemic dietary patterns; EDIP, empirical dietary inflammatory pattern score assessing the ability of the dietary pattern to contribute to chronic systemic inflammation - higher EDIP scores reflect more pro-inflammatory dietary patterns; HEI-2015, healthy eating index-2015 assessing adherence to the 2015-2020 Dietary Guidelines for Americans - higher HEI-2015 scores are indicative of greater adherence and higher dietary quality. EDIH and EDIP are positively correlated, whereas both scores are inversely correlated with HEI-2015, i.e., more hyperinsulinemic or pro-inflamamtory dietary patterns are of lower dietary quality. Each dietary score was adjusted for total energy intake using the residual method.

d.We tested for interaction using the likelihood ratio test, comparing the full (with dietary score x BMI terms) and reduced models (without interaction terms).

e. Due to small sample size, we combined the overweight and obese categories into one.

f. Data are not available because of smaller sample sizes for ER- PR- HER2+ subtype of the breast, non endometrioid endometrial cancer, ovarian cancer and non small cell lung cancer.

Supplementary Table S7. Multivariable-adjusted associations of dietary patterns with specific cancers in type 2 diabetes subgroups[a,b,c]

	1-SD increment in dietary score	P-value	1-SD increment in dietary score	P-value	P interaction[d]
	Had type 2 diabetes		*Did not have type 2 diabetes*		
**Total cancer (except non-melanoma skin cancer)**					
EDIH	1.02 (0.95, 1.09)	0.6422	**1.02 (1.01, 1.04)**	0.0072	1.0000
EDIP	1.03 (0.94, 1.13)	0.5700	**1.03 (1.01, 1.06)**	0.002	0.7642
HEI2015	1.00 (0.93, 1.07)	0.9673	**0.97 (0.95, 0.98)**	<.0001	0.3623
**Colorectal cancer**					
EDIH	1.16 (0.93, 1.44)	0.2007	**1.06 (1.01, 1.13)**	0.029	0.8821
EDIP	1.13 (0.81, 1.56)	0.4729	**1.09 (1.01, 1.17)**	0.0295	0.3383
HEI2015	0.97 (0.76, 1.24)	0.8304	**0.92 (0.87, 0.98)**	0.0043	0.6810
**Colon Cancer**					
EDIH	1.11 (0.86, 1.44)	0.4191	**1.08 (1.02, 1.15)**	0.0152	0.8769
EDIP	1.10 (0.77, 1.58)	0.5981	**1.11 (1.02, 1.20)**	0.0155	0.5947
HEI2015	0.94 (0.72, 1.23)	0.6662	**0.92 (0.86, 0.97)**	0.0036	0.6976
Proximal colon cancer					
EDIH	1.07 (0.76, 1.50)	0.7135	**1.12 (1.04, 1.21)**	0.0046	0.5174
EDIP	0.92 (0.58, 1.44)	0.7035	**1.09 (0.98, 1.22)**	0.0949	0.2626
HEI2015	1.09 (0.76, 1.57)	0.6365	0.92 (0.86, 1.00)	0.0422	0.4382
Distal colon and rectal cancer					
EDIH	1.11 (0.80, 1.54)	0.5275	1.04 (0.94, 1.14)	0.4614	0.8495
EDIP	1.40 (0.88, 2.23)	0.16	1.05 (0.98, 1.25)	0.1178	0.9128
HEI2015	1.10 (0.78, 1.57)	0.5854	**0.91 (0.84, 1.00)**	0.0454	0.2800
Intestinal polyps					
EDIH	0.99 (0.90, 1.09)	0.851	**1.08 (1.06, 1.10)**	<.0001	0.0261
EDIP	0.95 (0.84, 1.08)	0.4368	**1.07 (1.04, 1.10)**	<.0001	0.0886
HEI2015	0.97 (0.88, 1.07)	0.5502	**0.93 (0.92, 0.95)**	<.0001	0.2367
**Invasive breast cancer[e]**					
EDIH	0.92 (0.79, 1.06)	0.2487	**1.05 (1.02, 1.09)**	0.0035	0.1352
EDIP	1.10 (0.90, 1.34)	0.3417	**1.05 (1.00, 1.09)**	0.04	0.6330
HEI2015	1.05 (0.90, 1.22)	0.5283	0.99 (0.96, 1.02)	0.4777	0.4223
ER+					
EDIH	0.99 (0.85, 1.18)	0.9854	**1.04 (1.00, 1.08)**	0.0579	0.6421
EDIP	1.05 (0.84, 1.33)	0.6529	1.03 (0.98, 1.08)	0.2181	0.8993
HEI2015	1.04 (0.87, 1.24)	0.6593	1.00 (0.96, 1.04)	0.9768	0.5071
ER-					
EDIH	**0.68 (0.46, 0.98)**	0.0417	**1.14 (1.04, 1.25)**	0.0054	0.0282
EDIP	1.34 (0.85, 2.09)	0.2064	**1.10 (0.97, 1.25)**	0.1384	0.3608
HEI2015	1.00 (0.70, 1.42)	0.9882	0.95 (0.87, 1.04)	0.3041	0.8744
PR+					
EDIH	1.02 (0.85, 1.22)	0.8287	**1.05 (1.01, 1.09)**	0.025	0.8537
EDIP	1.02 (0.80, 1.32)	0.8581	1.04 (0.98, 1.09)	0.1882	0.9383
HEI2015	1.03 (0.85, 1.24)	0.7925	0.99 (0.95, 1.03)	0.6974	0.6235
PR-					
EDIH	**0.75 (0.56, 1.00)**	0.0474	**1.07 (1.00, 1.14)**	0.0546	**0.0287**

EDIP	1.25 (0.88, 1.77)	0.2203	1.05 (0.95, 1.15)	0.3372	0.5033
HEI2015	1.11 (0.84, 1.47)	0.4783	0.98 (0.92, 1.05)	0.6485	0.4620
HER2+					
EDIH	0.95 (0.60, 1.51)	0.8364	**1.10 (0.99, 1.22)**	0.0811	0.6315
EDIP	1.19 (0.61, 2.05)	0.7156	1.09 (0.95, 1.26)	0.2227	0.8003
HEI2015	0.70 (0.45, 1.10)	0.1259	0.99 (0.89, 1.10)	0.8166	0.1112
HER2-					
EDIH	0.96 (0.81, 1.13)	0.6034	**1.04 (1.00, 1.08)**	0.0353	0.3929
EDIP	1.09 (0.86, 1.37)	0.4719	**1.04 (0.98, 1.09)**	0.1708	0.7133
HEI2015	1.06 (0.89, 1.27)	0.4937	0.99 (0.96, 1.03)	0.6956	0.2863
Luminal A					
EDIH	0.99 (0.83, 1.18)	0.9069	1.03 (0.98, 1.07)	0.2139	0.7720
EDIP	1.09 (0.84, 1.40)	0.523	1.03 (0.97, 1.08)	0.3258	0.6360
HEI2015	1.08 (0.90, 1.32)	0.4044	0.99 (0.95, 1.03)	0.7043	0.1777
Luminal B					
EDIH	1.10 (0.66, 1.86)	0.7138	**1.14 (1.01, 1.29)**	0.0358	0.5281
EDIP	1.07 (0.53, 2.17)	0.852	1.10 (0.94, 1.30)	0.2561	0.4092
HEI2015	0.64 (0.38, 1.08)	0.0971	1.02 (0.91, 1.16)	0.7118	**0.0719**
Triple negative					
EDIH	0.79 (0.51, 1.23)	0.2979	**1.19 (1.06, 1.33)**	0.0035	**0.0790**
EDIP	1.12 (0.65, 1.94)	0.6807	1.11 (0.94, 1.30)	0.2108	0.6976
HEI2015	0.94 (0.61, 1.46)	0.7858	0.98 (0.87, 1.10)	0.7087	0.7205
Invasive ductal carcinoma					
EDIH	0.89 (0.74, 1.08)	0.248	**1.06 (1.01, 1.11)**	0.011	**0.0771**
EDIP	1.10 (0.85, 1.42)	0.4649	**1.04 (0.98, 1.10)**	0.1577	0.9287
HEI2015	1.09 (0.90, 1.33)	0.368	1.01 (0.96, 1.06)	0.6975	0.3097
Localized					
EDIH	0.88 (0.73, 1.05)	0.143	**1.07 (1.03, 1.11)**	0.001	**0.0299**
EDIP	1.05 (0.83, 1.33)	0.6981	**1.05 (1.00, 1.10)**	0.0545	0.6938
HEI2015	1.13 (0.94, 1.36)	0.1846	0.99 (0.96, 1.03)	0.7772	0.1094
Regional/distant					
EDIH	1.02 (0.78, 1.34)	0.8752	1.01 (0.94, 1.08)	0.869	0.5639
EDIP	1.20 (0.82, 1.75)	0.3467	1.02 (0.94, 1.12)	0.6334	0.2963
HEI2015	0.87 (0.65, 1.17)	0.3546	0.99 (0.92, 1.06)	0.7244	0.3097
**Endometrial cancer**					
EDIH	1.00 (0.68, 1.45)	0.9833	**1.12 (1.00, 1.24)**	0.0432	0.5381
EDIP	1.17 (0.70, 1.96)	0.5457	**1.13 (0.98, 1.31)**	0.0958	0.8395
HEI2015	0.94 (0.64, 1.37)	0.7509	0.96 (0.86, 1.07)	0.45	0.8963
**Lung cancer**					
EDIH	1.01 (0.81, 1.27)	0.9109	0.95 (0.90, 0.99)	0.0255	**0.0479**
EDIP	1.18 (0.86, 1.63)	0.2978	0.96 (0.90, 1.02)	0.1876	**0.0242**
HEI2015	**0.76 (0.60, 0.96)**	0.0189	0.97 (0.93, 1.02)	0.2258	**0.0175**

a. HRs were derived from multivariable-adjusted Cox proportional hazards regression models adjusted for the following baseline covariates: age at enrollment, physical activity, race and ethnicity, educational level, family history of cancer, number of hormones used, comorbidity score, baseline cardiovascular disease status, baseline lung disease, number of supplements used, non-steroidal anti-inflammatory drug use, hormone therapy study arm (Not randomized to HRT, E-alone intervention, E-alone control, E+P intervention, E+P control), baseline hormone therapy ever, oral contraceptive duration, pack-years of smoking, coffee/tea, and total alcohol intake. Colorectal cancer and subtype analyses were additionally adjusted for colorectal cancer screening. Invasive breast cancer and subtype analyses were additionally adjusted for hysterectomy age, months of breast-feeding, age at menopause, mammogram ever, parity, bilateral oophorectomy, passive smoking, Gail 5-year risk score. Endometrial cancer and ovarian cancer analyses were additionally adjusted for age at first birth, age at menarche, age at menopause, months of breast-feeding, and parity. Ovarian cancer analyses were further adjusted for tubal ligation. Lung cancer analyses were additionally adjusted for smoking status (current, former, never smokers) and passive smoking. Dietary score was input in each model as a continuous variable.

b. We presented here the cancer sites that were found to be associated with dietary patterns in the main analysis. Cancer sites were defined as follows: total cancer was the first occurrence of any cancer except non-melanoma skin cancer. Proximal colon - cecum, ascending colon, hepatic flexure of colon, transverse colon (ICD site codes 18.0,18.2,18.3, 18.4); distal colon - splenic flexure of colon, descending colon, sigmoid colon (ICD site codes 18.5, 18.6,18.7); Luminal A breast cancer - ER+ and/or PR+, HER2-; luminal B breast cancer - ER+ and/or PR+ , HER2+.

c. EDIH, empirical dietary index for hyperinsulinemia score assessing the ability of the dietary pattern to contribute to insulin hypersecretion - higher EDIH scores reflect more hyperinsulinemic dietary patterns; EDIP, empirical dietary inflammatory pattern score assessing the ability of the dietary pattern to contribute to chronic systemic inflammation - higher EDIP scores reflect more pro-inflammatory dietary patterns; HEI-2015, healthy eating index-2015 assessing adherence to the 2015-2020 Dietary Guidelines for Americans - higher HEI-2015 scores are indicative of greater adherence and higher dietary quality. EDIH and EDIP are positively correlated, whereas both scores are inversely correlated with HEI-2015, i.e., more hyperinsulinemic or pro-inflamamtory dietary patterns are of lower dietary quality. Each dietary score was adjusted for total energy intake using the residual method.

d.We tested for interaction using the likelihood ratio test, comparing the full (with dietary score x type II diabetes status terms) and reduced models (without interaction terms).

e.Data are not available because of smaller sample sizes for invasive luminal carcinoma and ER- PR- HER2+ subtypes of the breast, endometrial cancer subtypes, ovarian cancer and small cell lung cancer.

Supplementary Table S8. Multivariable-adjusted associations of dietary patterns with specific cancers including mutual adjustment[a]

	Q1	Q2	Q3	Q4	Q5	1-SD increment in dietary score	P-value
**Total cancer**							
Cases/ noncases	3934/ 18559	3861/ 18633	3772/ 18722	3594/ 18900	3607/ 18886		
EDIH	1 (ref)	1.04 (0.99, 1.09)	1.05 (1.00, 1.10)	1.03 (0.98, 1.08)	**1.07(1.01, 1.13)**	**1.01 (0.99, 1.03)**	0.3489
	4131/18362	4027/18467	3703/18791	3608/18886	3299/19194		
EDIP	1 (ref)	1.04 (0.99, 1.09)	1.00 (0.95, 1.05)	1.03 (0.97, 1.10)	1.03 (0.96, 1.10)	1.02 (0.99, 1.04)	0.1623
	3697/18796	3740/18753	3786/18708	3750/18743	3795/18698		
HEI-2015	1 (ref)	0.98 (0.94, 1.03)	0.98 (0.93, 1.02)	0.95 (0.90, 1.00)	0.95 (0.90, 1.00)	**0.97 (0.96, 0.99)**	**0.0017**
**Colorectal cancer**							
Cases/ noncases	301/ 21146	309/ 21139	372/ 21075	306/ 21142	320/ 21127		
EDIH	1 (ref)	1.04 (0.87, 1.23)	1.26 (1.06, 1.49)	1.02 (0.85, 1.23)	1.10 (0.90, 1.34)	1.03 (0.97, 1.10)	0.3447
	315/21132	333/21115	338/21109	319/21129	303/21144		
EDIP	1 (ref)	1.08 (0.91, 1.29)	1.11 (0.92, 1.34)	1.10 (0.89, 1.35)	1.11 (0.88, 1.41)	1.03 (0.95, 1.13)	0.4446
	342/21105	337/21110	316/21131	297/21150	316/21131		
HEI-2015	1 (ref)	0.97 (0.83, 1.13)	0.91 (0.78, 1.07)	0.84 (0.71, 0.99)	0.88 (0.74, 1.05)	0.95 (0.90, 1.00)	0.0564
**Colon cancer**							
Cases/ noncases	250/ 21217	264/ 21203	312/ 21155	254/ 21213	266/ 21201		
EDIH	1 (ref)	1.06 (0.88, 1.28)	1.27 (1.06, 1.54)	1.02 (0.84, 1.25)	1.10 (0.88, 1.36)	1.04 (0.96, 1.11)	0.3285
	260/21207	286/21181	279/21188	266/21201	255/21212		
EDIP	1 (ref)	1.13 (0.93, 1.36)	1.11 (0.90, 1.37)	1.12 (0.88, 1.40)	1.16 (0.89, 1.50)	1.04 (0.95, 1.15)	0.3715
	285/21181	284/21183	264/21203	249/21218	264/21202		
HEI-2015	1 (ref)	0.97 (0.82, 1.15)	0.91 (0.76, 1.08)	0.83 (0.69, 0.99)	0.86 (0.71, 1.04)	0.94 (0.88, 1.00)	0.0534
**Invasive breast cancer**							
Cases/ noncases	899/ 17565	921/ 17544	827/ 17637	843/ 17622	903/ 17561		
EDIH	1 (ref)	1.08 (0.98, 1.19)	1.01 (0.91, 1.12)	1.06 (0.95, 1.18)	**1.18 (1.05, 1.33)**	**1.03 (1.00, 1.08)**	0.0857
	926/17538	936/17529	874/17590	866/17599	791/17673		
EDIP	1 (ref)	1.08 (0.97, 1.19)	1.05 (0.94, 1.17)	1.09 (0.97, 1.24)	1.05 (0.91, 1.20)	1.03 (0.98, 1.08)	0.2557
	872/17592	867/17597	847/17617	869/17595	938/17526		
HEI-2015	1 (ref)	0.96 (0.87, 1.06)	0.93 (0.84, 1.02)	0.95 (0.86, 1.06)	1.04 (0.94, 1.15)	1.01 (0.97, 1.04)	0.6743
**Endometrial cancer**							
Cases/ noncases	74/ 8946	88/ 8932	79/ 8942	73/ 8947	89/ 8931		
EDIH	1 (ref)	1.30 (0.94, 1.80)	1.22 (0.86, 1.73)	1.20 (0.83, 1.74)	**1.54 (1.05, 2.26)**	1.09 (0.97, 1.23)	0.1680
	81/8939	93/8927	67/8954	84/8936	78/8942		
EDIP	1 (ref)	1.12 (0.80, 1.57)	0.84 (0.57, 1.24)	1.10 (0.73, 1.66)	1.14 (0.72, 1.80)	1.09 (0.93, 1.28)	0.2872
	67/8953	84/8936	98/8923	81/8939	73/8947		
HEI-2015	1 (ref)	1.21 (0.87, 1.68)	1.36 (0.99, 1.88)	1.11 (0.79, 1.57)	1.00 (0.69, 1.44)	1.00 (0.89, 1.11)	0.9364
**Lung cancer**							
Cases/ noncases	482/ 20722	408/ 20797	410/ 20795	417/ 20788	399/ 20805		
EDIH	1 (ref)	0.93 (0.81, 1.07)	0.95 (0.82, 1.10)	0.98 (0.84, 1.14)	0.85 (0.72, 1.00)	0.94 (0.90, 1.00)	0.0422
	547/20657	464/20741	397/20808	377/20828	331/20873		
EDIP	1 (ref)	1.06 (0.92, 1.22)	0.99 (0.84, 1.16)	1.05 (0.88, 1.25)	1.05 (0.86, 1.28)	0.99 (0.92, 1.06)	0.6728
	520/20684	439/20765	415/20789	387/20817	354/20850		

HEI-2015	1 (ref)	0.97 (0.85, 1.10)	0.98 (0.85, 1.12)	0.95 (0.82, 1.09)	0.88 (0.76, 1.03)	0.94 (0.89, 0.98)	0.0090

a.All three dietary indices were included in the same multivariable adjusted model. Values presented are hazard ratios (HR) and 95% confidence intervals (95% CI) for relative risk and incidence rate per 100,000 person-years for absolute risk. HRs were derived from multivariable-adjusted Cox proportional hazards regression models adjusted for the following baseline covariates: age at enrollment, physical activity, race and ethnicity, educational level, family history of cancer, number of hormones used, comorbidity score, baseline cardiovascular disease status, baseline lung disease, number of supplements used, non-steroidal anti-inflammatory drug use, hormone therapy study arm, baseline hormone therapy ever, oral contraceptive duration, pack-years of smoking, coffee/tea, and total alcohol intake. Colorectal cancer and subtype analyses were additionally adjusted for colorectal cancer screening. Invasive breast cancer and subtype analyses were additionally adjusted for months of breast-feeding, age at menopause, mammogram ever, parity, bilateral oophorectomy, passive smoking, Gail 5-year risk score. Endometrial cancer and ovarian cancer analyses were additionally adjusted for age at first birth, age at menarche, age at menopause, months of breast-feeding, and parity. Ovarian cancer analyses were further adjusted for tubal ligation. Lung cancer analyses were additionally adjusted for smoking status and passive smoking.
